# Supplementary material for: A new form of diabetes caused by INS mutations defined by zygosity, stem cell and population data
Source: EMBO Mol Med. 2026 Jan 3;18(2):620–45. doi: 10.1038/s44321-025-00362-9 (PMC12905373; doi:10.1038/s44321-025-00362-9)
Supplement: Supplementary file 1 — Appendix [file 44321_2025_362_MOESM1_ESM.pdf]

Appendix

Table of Contents

Appendix *1*

Appendix figures and legends 2

Appendix Figure S1. Pedigrees of published *INS* R6C and R6H families 3

Appendix Figure S2. Complementary expression of *INS* R6C mutant in insulin knockout EndoC-βH1 cells does not induce cell death 4

Appendix Figure S3. Generation of heterozygous R6C and corrected iPSC lines 5

Appendix Figure S4. Karyotyping of heterozygous R6C and corrected iPSC lines 6

Appendix Figure S6. Gene expression along the differentiation of heterozygous R6C and corrected iPSCs into islets 8

Appendix Figure S7. Generation of homozygous R6C and corrected iPSC lines 9

Appendix Figure S8. Karyotyping of homozygous R6C iPSC lines 10

Appendix Figure S9. Karyotyping of homozygous corrected R6C iPSC lines 11

Appendix Figure S10. Quality control of homozygous R6C and corrected iPSC lines 12

Appendix Figure S11. Gene expression along the differentiation of homozygous R6C and corrected iPSCs into islets 13

Appendix Figure S12. Seahorse mitochondrial respiration profile in homozygous R6C and corrected cells. 14

Appendix Figure S13. *In vivo* maturation of homozygous R6C and corrected β cells and GLP-1RA *in vitro* and *in vivo* treatment 15

Appendix Figure S14. Normal signaling of insulin secreted from R6C mutant and isogenic corrected iPSC-islets 16

Appendix Figure S15. R6C *INS* does not induce ER stress 17

Appendix Figure S16. No worsening of ER stress in homozygous R6C β cells by ER stressors and GLP1-RAs 18

Appendix Figure S17. Protection against apoptosis in homozygous R6C β cells by GLP1-RAs 20

Appendix tables and legends 21

Appendix Table S1. Allele frequency of *INS* R6C in population-level datasets 22

Appendix Table S2. *In silico* prediction of the *INS* R6C mutation 22

Appendix Table S3. CRISPR/Cas9 editing and on-target and off-target profiling on pre- and post-CRISPR iPSCs 23

Appendix Table S4. *In silico* prediction of structure and wild type and *INS* R6C signal peptide interaction with SRP54 and SEC61α2 24

Appendix Table S5. Differentially expressed genes between corrected and homozygous R6C β cell-purified aggregates 25

Appendix Table S6. Custom curated gene-wise z-scores of key β cell function pathways27

Appendix Table S7. qPCR primers 28

Appendix Table S8. Antibodies 30

Appendix figures and legends

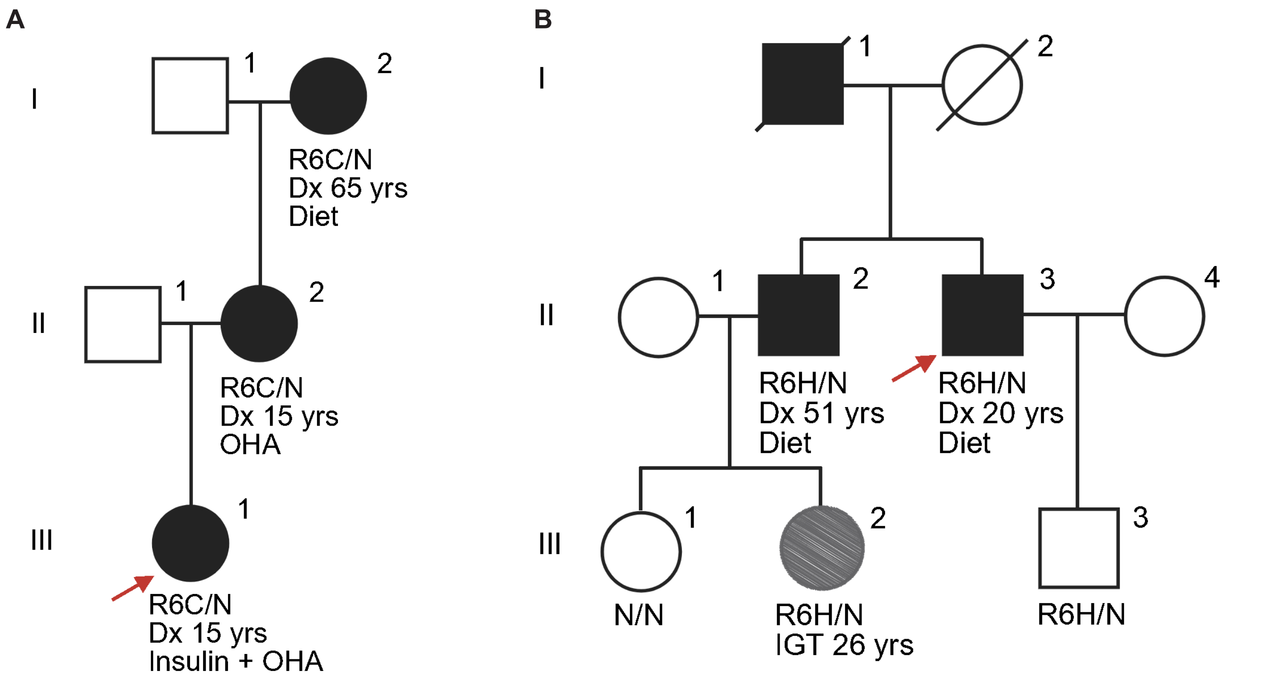

Appendix Figure S1. Pedigrees of published *INS* R6C and R6H families

(A) The first reported *INS* c.16C>T, p.(Arg6Cys), R6C family (Edghill *et al*, 2008). (B) The first reported *INS* c.17G>A, p.(Arg6His), R6H family (Meur *et al*, 2010). Red arrow indicates the probands. Black solid symbol indicates diagnosis of diabetes, void symbol indicates unaffected individual, gray shaded indicates impaired glucose tolerance (IGT), and crossed symbol indicates deceased. Roman numerals show generations and Arabic numerals individuals within each generation. R6C/R6C: homozygous, R6C/N or R6H/N: heterozygous. N/N: non-carrier; other individuals lack genetic confirmation. Dx: age at diagnosis of diabetes; Diet: diet control; OHA: oral hypoglycemic agents; insulin: insulin treatment.

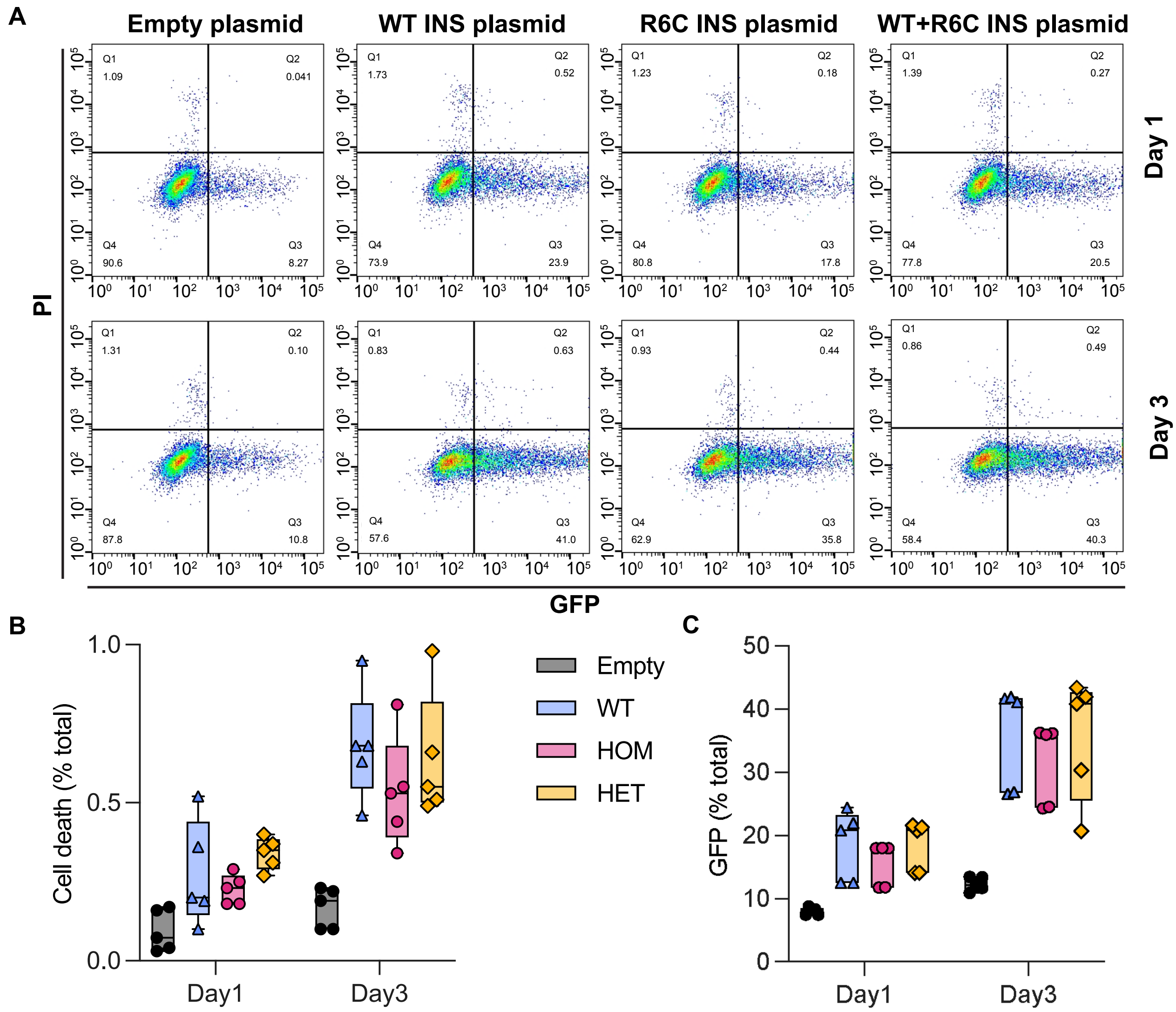

Appendix Figure S2. Complementary expression of *INS* R6C mutant in insulin knockout EndoC-βH1 cells does not induce cell death

EndoC-βH1 INS-knockout cells were transfected with plasmids expressing wildtype (blue, WT) insulin, 100% R6C insulin (pink, R6C), 50% R6C + 50% wildtype insulin (yellow, HET), or GFP only (black, Empty) for 1 or 3 days (n=5 for each). Cells were stained with propidium iodide (PI) and sorted for GFP expression and PI staining. (A) Representative flow cytometry dot plots. The x-axis indicates GFP, and the y-axis PI fluorescence intensity. The percentage of cells in each quadrant is indicated. Data are representative of n=5 independent experiments. (B) Quantification of β cell death (%) as measured by double positivity for GFP and PI in total cell population. (C) Quantification of transfection efficiency (%) as measured by percentage of total GFP positivity. In box plots, the median of independent experiments is shown by a horizontal line; 25th and 75th percentiles are at the bottom and top of the boxes; whiskers represent minimum and maximum values.

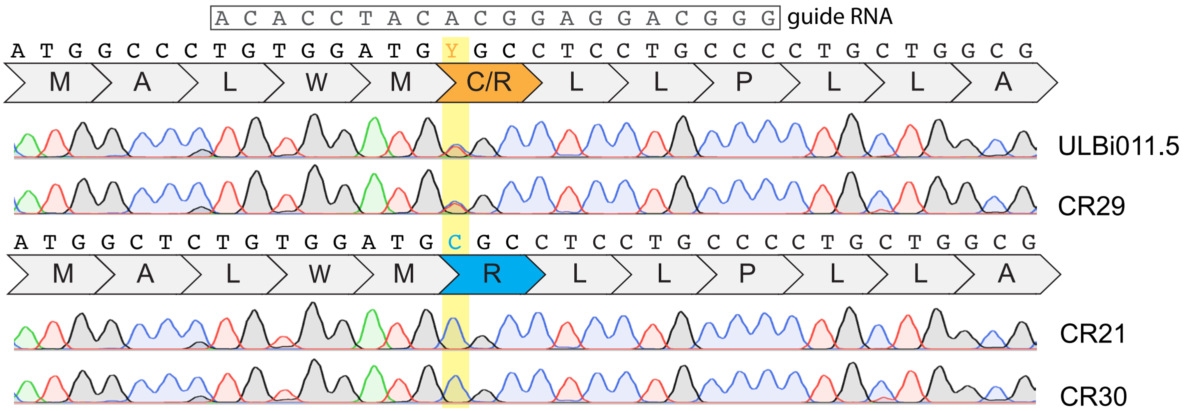

Appendix Figure S3. Generation of heterozygous R6C and corrected iPSC lines

(A) DNA and amino acid sequences for the first 12 codons of *INS*. The yellow shadow highlights the NM_001185098.1:c.16C>T heterozygous (C/T, yellow) and wild-type variant (C, blue). Top: Reference sequence and Sanger sequencing chromatogram of heterozygous R6C iPSC line ULBi011.5 and CRISPRed ULBi011.5 CR29. Bottom: Homology directed repair template sequence and Sanger sequencing chromatogram of corrected iPSC lines ULBi011.5 CR21 and CR30.

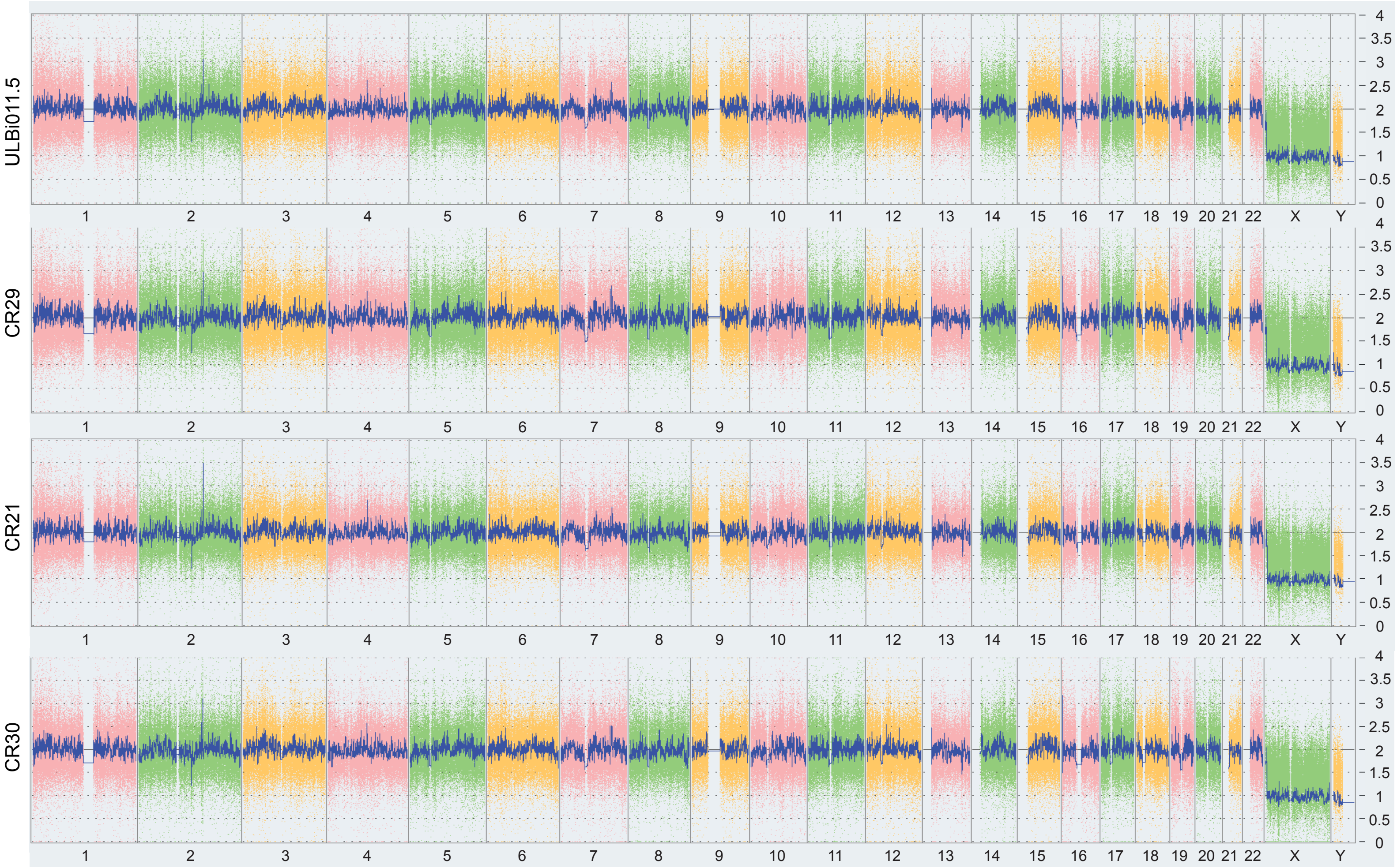

Appendix Figure S4. Karyotyping of heterozygous R6C and corrected iPSC lines

KaryoStat of iPSC lines ULBi011.5, ULBi011.5 CR29, CR21 and CR30. The whole genome view displays all somatic and sex chromosomes in one frame with high level copy number. The smooth signal plot (right y-axis) is the smoothing of the log_2 ratios which depict the signal intensities of probes on the microarray. A value of 2 represents a normal copy number state (CN = 2). A value of 3 represents chromosomal gain (CN = 3). A value of 1 represents a chromosomal loss (CN = 1). The pink, green and yellow colors indicate the raw signal for each individual chromosome probe, while the blue signal represents the normalized probe signal which is used to identify copy number and aberrations (if any). The KaryoStat analysis revealed no chromosomal aberration.

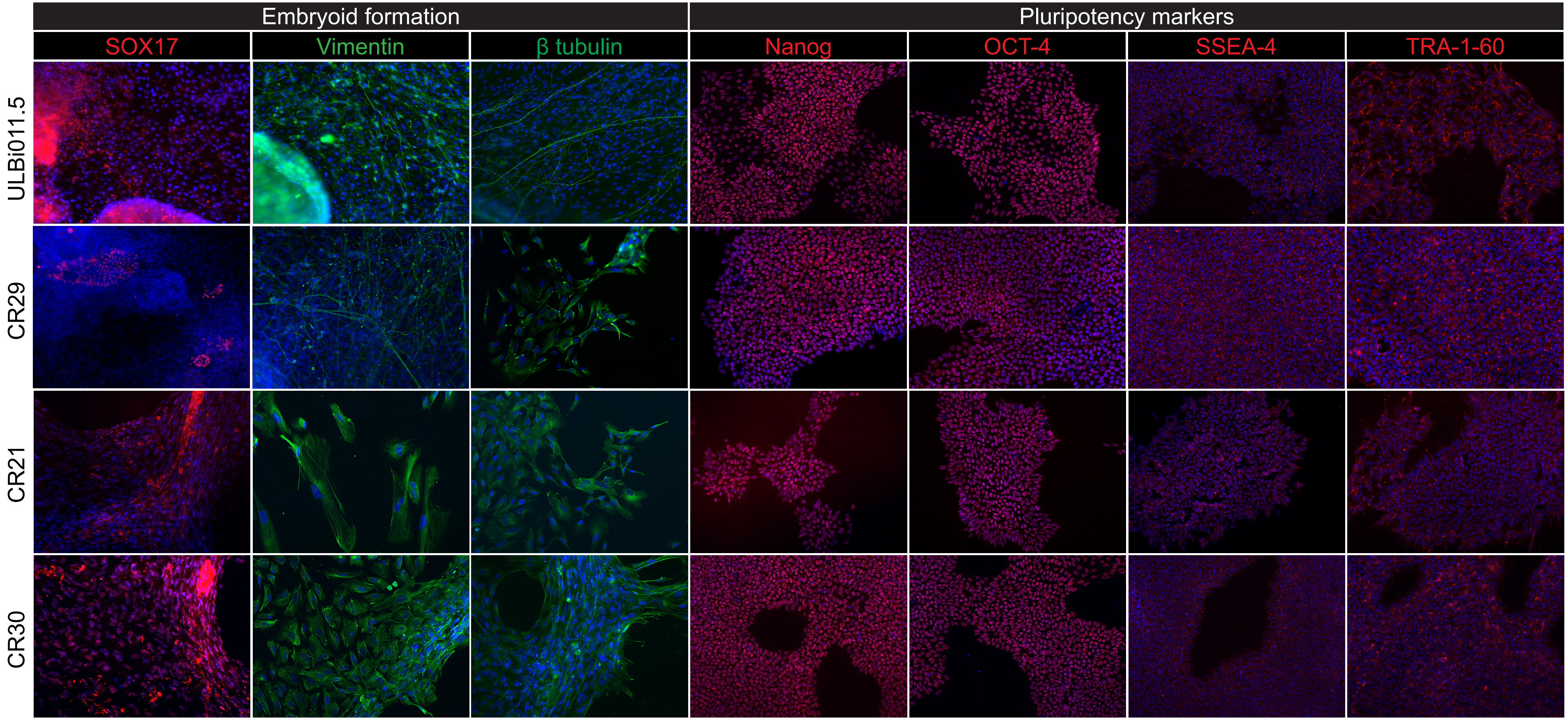

Appendix Figure S5. Quality control of heterozygous R6C and corrected iPSC lines

Embryoid body assay for the capability of iPSCs to differentiate into three germ layers endoderm, mesoderm and ectoderm, immunostained with SOX17 (red), Vimentin (green) and Beta Tubulin III (green), respectively. Immunostaining of iPSCs for pluripotency markers OCT4, SSEA4, Nanog and TRA1-60 (all in red). DAPI stains the nuclei in blue.

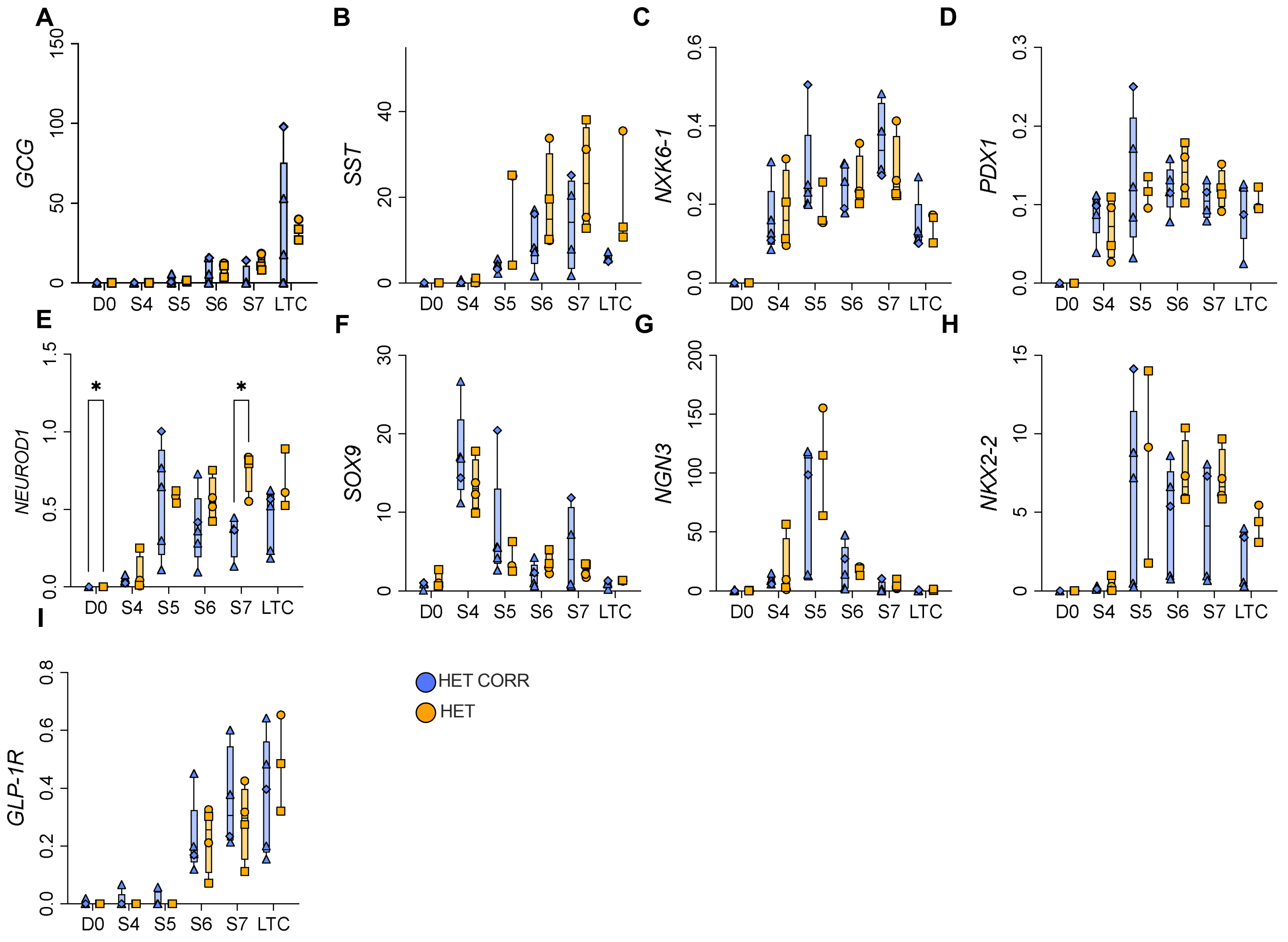

Appendix Figure S6. Gene expression along the differentiation of heterozygous R6C and corrected iPSCs into islets

(A-I) GCG, SST, NKX6-1, PDX1, NEUROD1, SOX9, NGN3, NKX2-2, and GLP-1R mRNA expression in heterozygous R6C and corrected iPSC lines at iPSC stage (D0) and along differentiation stages to long-term culture (LTC) following the differentiation protocol as shown in Figure EV3A. Data were normalized to the geometric mean of the reference genes β-Actin and VAPA. Sample sizes were HET CORR (n = 5) and HET (n = 4); individual stages contained missing observations. In box plots, the median of independent experiments is shown by a horizontal line; 25th and 75th percentiles are at the bottom and top of the boxes; whiskers represent minimum and maximum values.

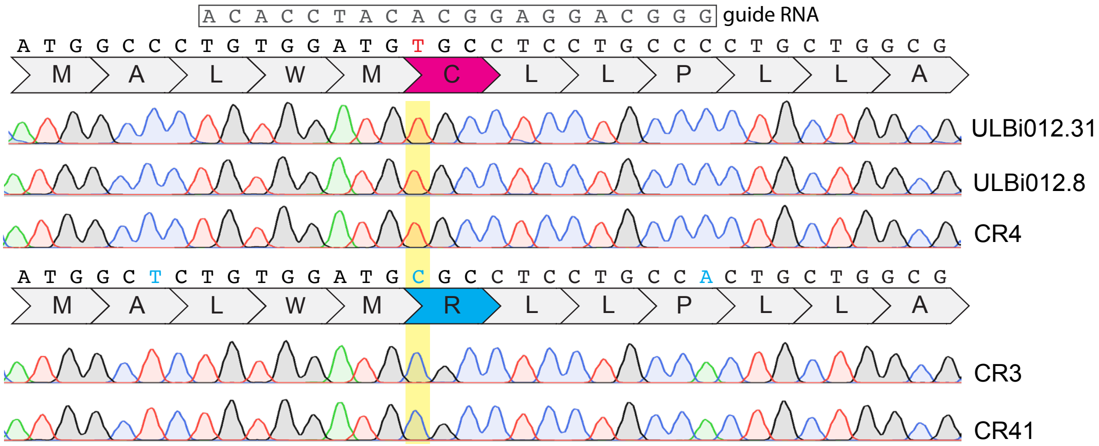

Appendix Figure S7. Generation of homozygous R6C and corrected iPSC lines

(A) DNA and amino acid sequences for the first 12 codons of *INS*. The yellow shadow highlights the NM_001185098.1:c.16C>T (red) and wild-type variant (C, blue). Top: Reference sequence and Sanger sequencing chromatogram of homozygous R6C iPSC lines ULBi012.31, ULBi012.8 and CRISPRed ULBi012.31 CR4. Bottom: Homology directed repair template sequence and Sanger sequencing chromatogram of corrected iPSC lines ULBi012.31 CR3 and CR41. The 6T and 27A (blue) indicate silent mutations disturbing PAM sequence and introducing a restriction site.

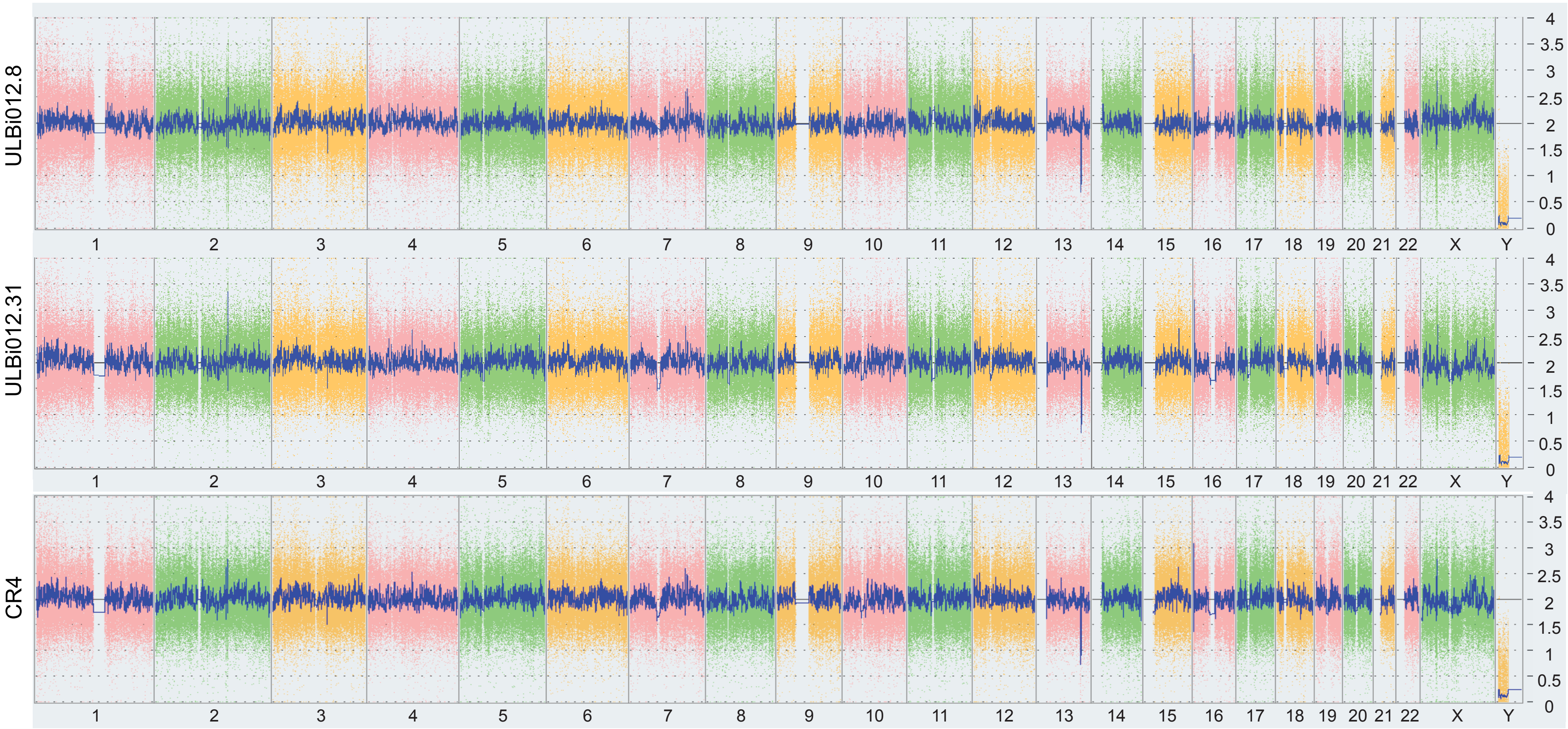

Appendix Figure S8. Karyotyping of homozygous R6C iPSC lines

KaryoStat of iPSC lines ULBi012.31, ULBi012.8, and ULBi012.31 CR4. The whole genome view displays all somatic and sex chromosomes in one frame with high level copy number. The smooth signal plot (right y-axis) is the smoothing of the log2 ratios which depict the signal intensities of probes on the microarray. A value of 2 represents a normal copy number state (CN = 2). A value of 3 represents chromosomal gain (CN = 3). A value of 1 represents a chromosomal loss (CN = 1). The pink, green and yellow colors indicate the raw signal for each individual chromosome probe, while the blue signal represents the normalized probe signal which is used to identify copy number and aberrations (if any). The KaryoStat analysis revealed no chromosomal aberration.

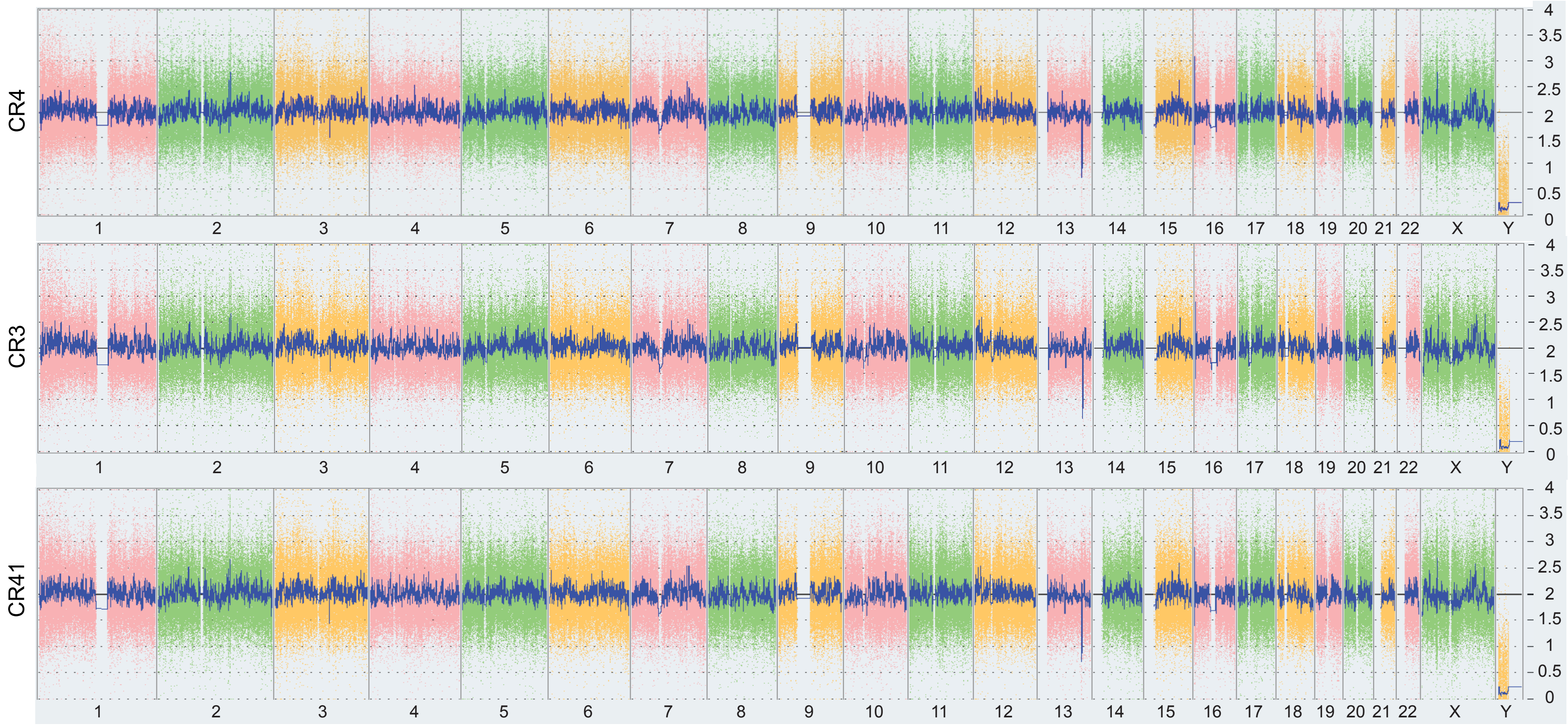

Appendix Figure S9. Karyotyping of homozygous corrected R6C iPSC lines

KaryoStat of iPSC lines ULBi012.31 CR3 and CR41. The whole genome view displays all somatic and sex chromosomes in one frame with high level copy number. The smooth signal plot (right y-axis) is the smoothing of the log2 ratios which depict the signal intensities of probes on the microarray. A value of 2 represents a normal copy number state (CN = 2). A value of 3 represents chromosomal gain (CN = 3). A value of 1 represents a chromosomal loss (CN = 1). The pink, green and yellow colors indicate the raw signal for each individual chromosome probe, while the blue signal represents the normalized probe signal which is used to identify copy number and aberrations (if any). The KaryoStat analysis revealed no chromosomal aberration.

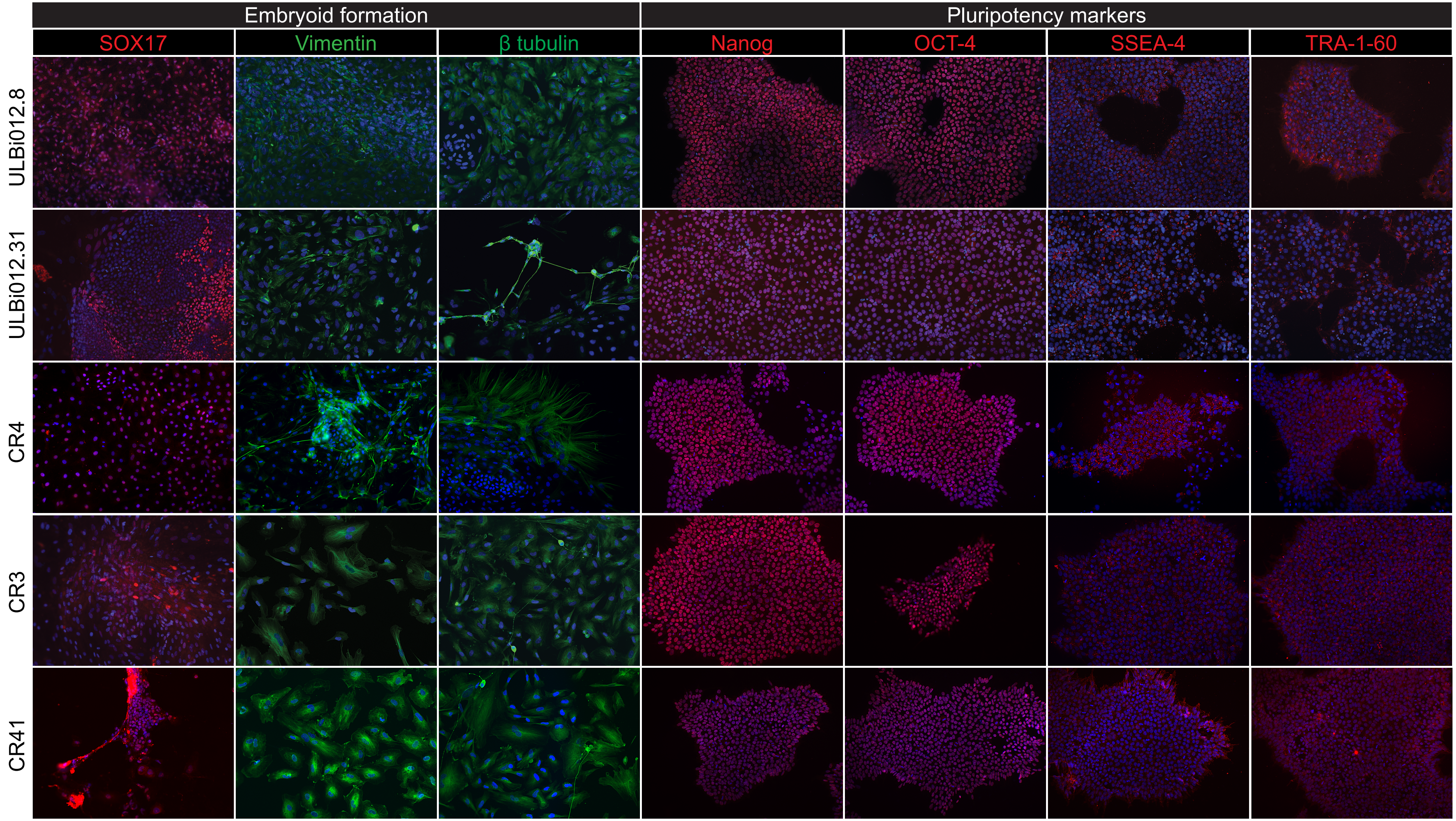

Appendix Figure S10. Quality control of homozygous R6C and corrected iPSC lines

Embryoid body assay for the capability of iPSCs to differentiate into three germ layers endoderm, mesoderm and ectoderm, immunostained with SOX17 (red), Vimentin (green) and Beta Tubulin III (green), respectively. Immunostaining of iPSCs for pluripotency markers OCT4, SSEA4, Nanog and TRA1-60 (all in red). DAPI stains the nuclei in blue.

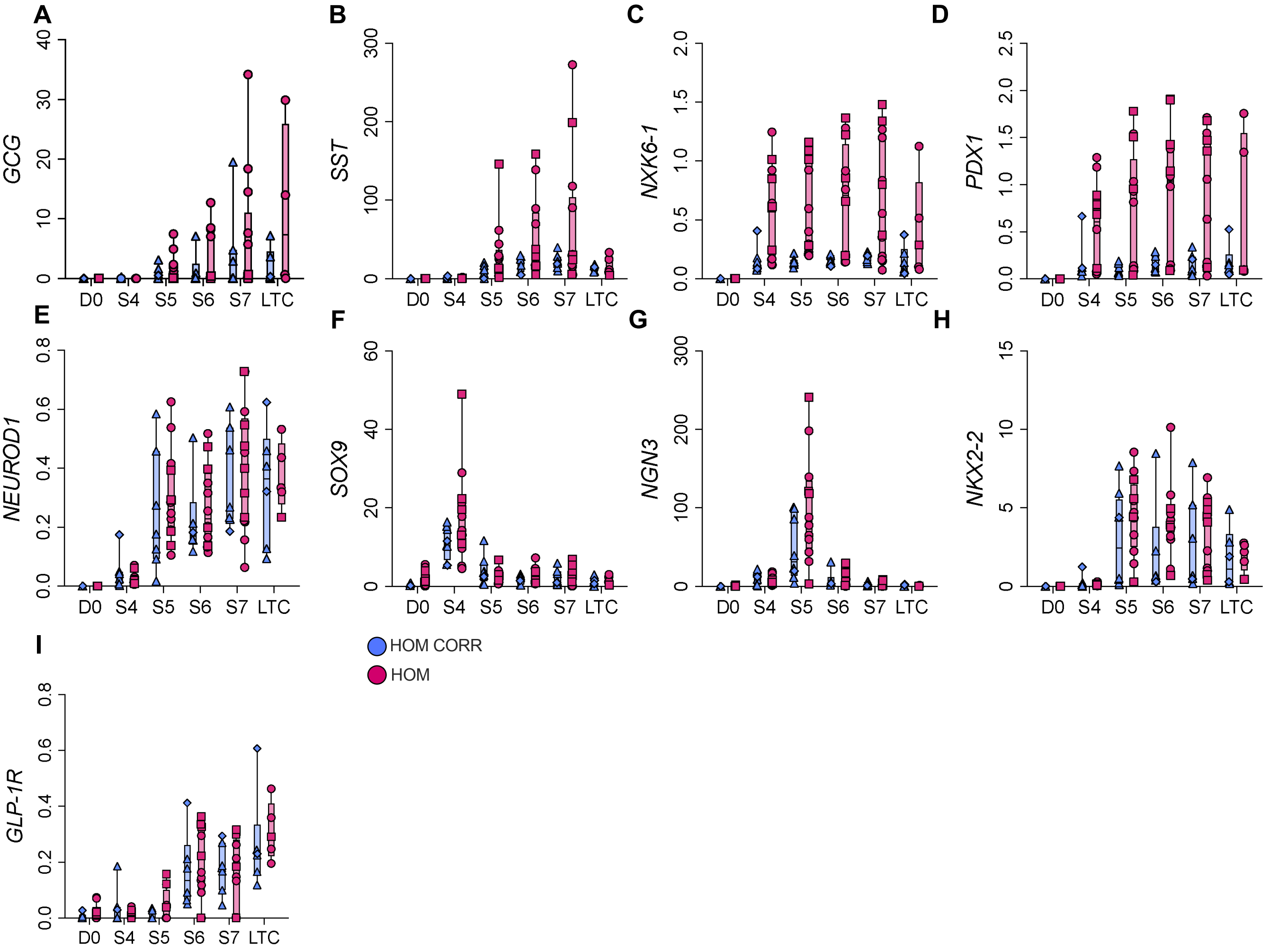

Appendix Figure S11. Gene expression along the differentiation of homozygous R6C and corrected iPSCs into islets

(A-I) GCG, SST, NKX6-1, PDX1, NEUROD1, SOX9, NGN3, NKX2-2, and GLP-1R mRNA expression in homozygous R6C and corrected iPSC lines at iPSC stage (D0) and along differentiation stages to long-term culture (LTC). Data were normalized to the geometric mean of the reference genes β-Actin and VAPA. Sample sizes were HOM CORR (n = 9) and HOM (n = 15); individual stages contained missing observations. In box plots, the median of independent experiments is shown by a horizontal line; 25th and 75th percentiles are at the bottom and top of the boxes; whiskers represent minimum and maximum values.

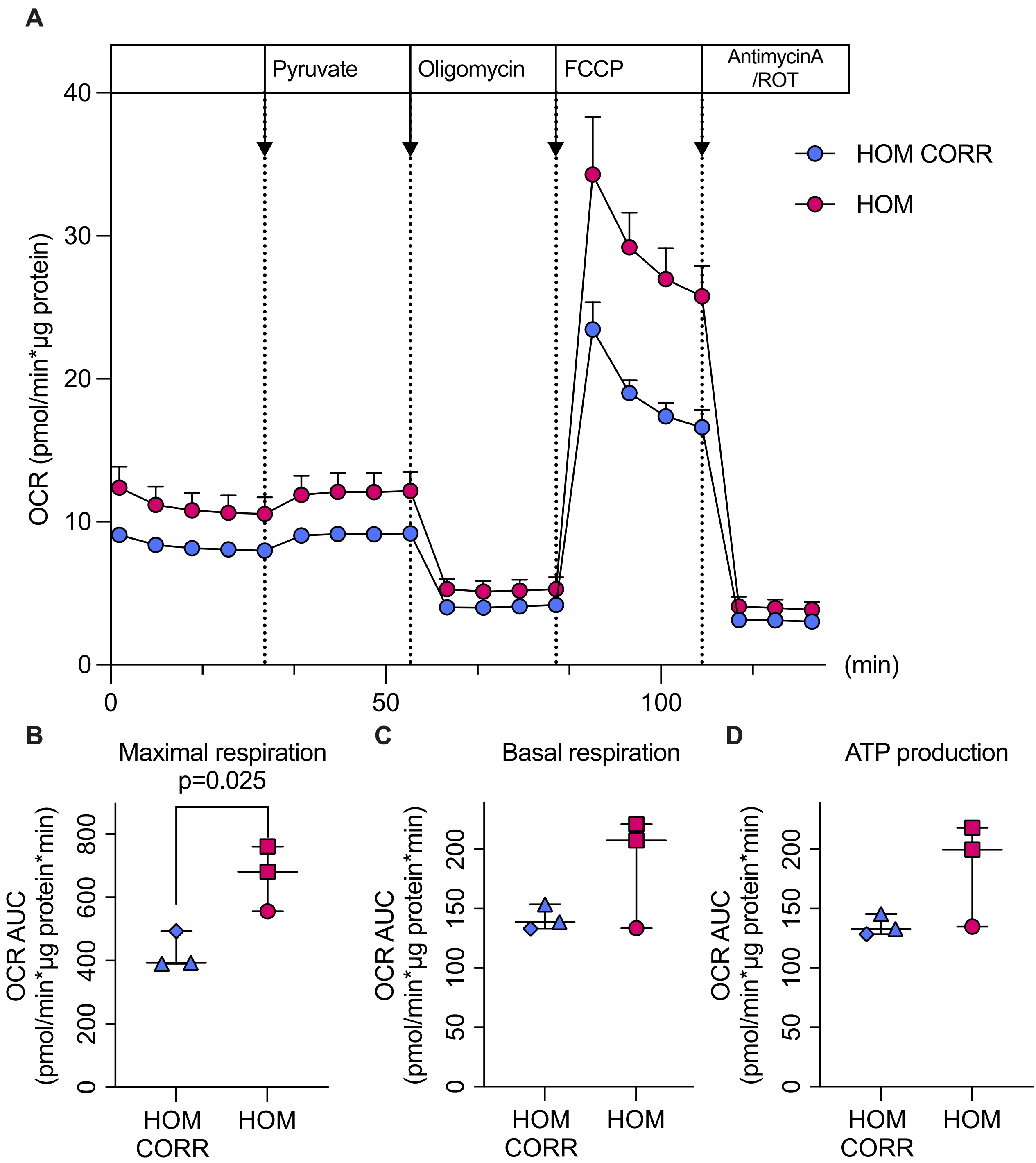

Appendix Figure S12. Seahorse mitochondrial respiration profile in homozygous R6C and corrected cells

(A) Oxygen consumption rate (OCR) over time in homozygous R6C (HOM, pink) and isogenic corrected (HOM CORR, blue) stage 7 iPSC-β cells following sequential injection of pyruvate, oligomycin, FCCP, and a combination of antimycin A and rotenone (ROT). Quantification of (B) maximal respiration, (C) basal respiration, and (D) ATP production derived from OCR area under the curve (AUC) calculations. HOM CORR n = 3, HOM n = 3. All panels: Unpaired t-test. In box plots, the median of independent experiments is shown by a horizontal line; 25th and 75th percentiles are at the bottom and top of the boxes; whiskers represent minimum and maximum values. In time course line plots, data are shown as mean ± s.e.m..

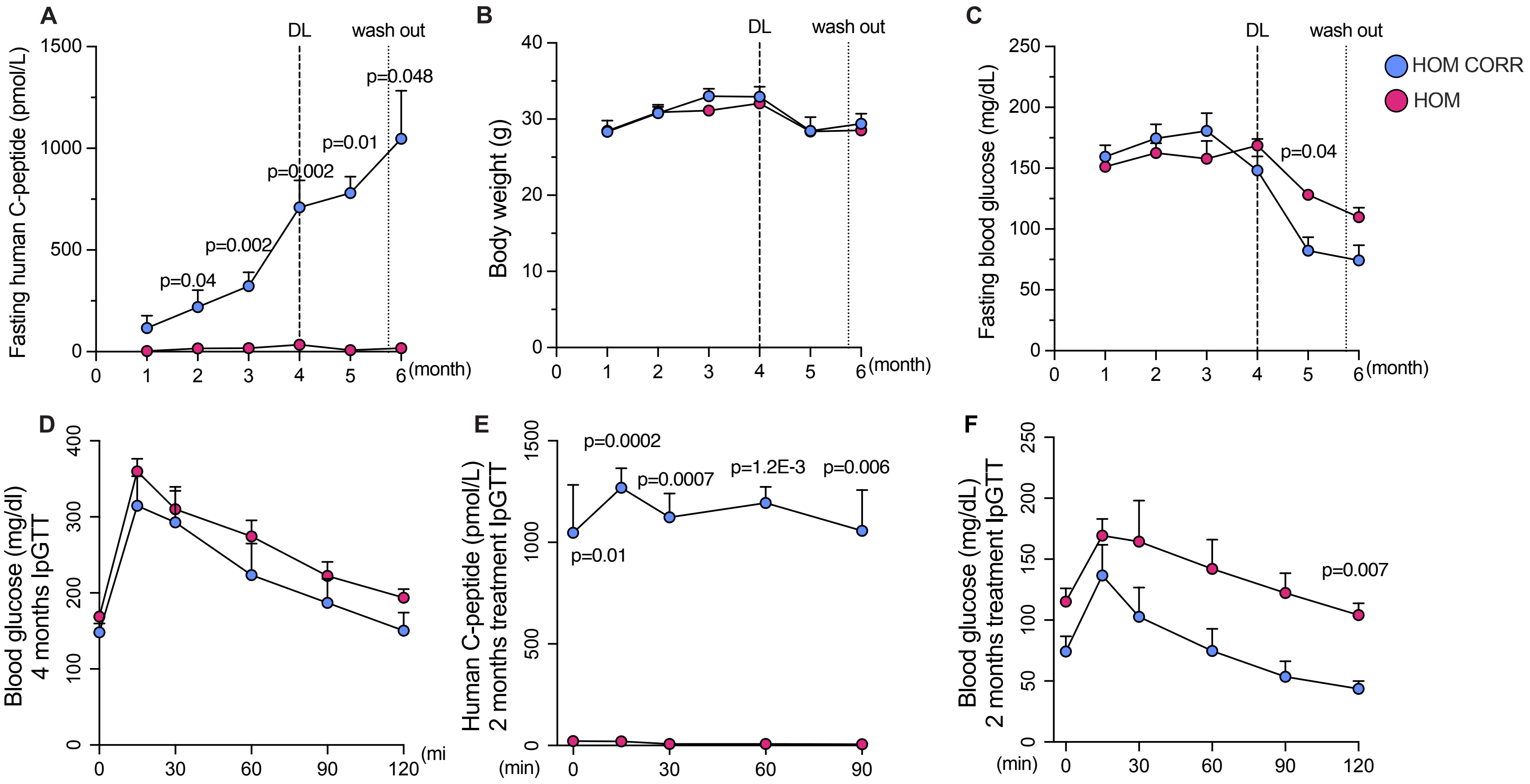

Appendix Figure S13. ***In vivo*** **maturation of homozygous R6C and corrected β cells and GLP-1RA** ***in vitro*** **and** ***in vivo*** **treatment**

(A-C) Homozygous R6C (HOM, pink) and isogenic corrected (HOM CORR, blue) MACS-purified iPSC-β cell aggregates were transplanted under the kidney capsule of immunocompromised Rag2 knockout mice and followed up for 4 months prior to a 2-month treatment with dulaglutide (DL, 1 mg/kg, twice weekly, dashed line). The last week (dotted line) represents washout of treatment. (A) Area under the curve (AUC) of fasting human C-peptide levels before intraperitoneal glucose tolerance test (IpGTT), (B) mouse body weight, and (C) fasting blood glucose levels from 1 month after transplantation to 2-month dulaglutide treatment. For (A), (B) and (C) mixed-effects analysis with Tukey correction for multiple comparison, sample sizes (n, HOM CORR vs. HOM) for each timepoint were: 1 month (7 vs. 17), 2 months (9 vs. 19), 3 months (7 vs. 17), 4 months (7 vs. 9), 5 months (3 vs. 8), 6 months (3 vs. 7). (D) Blood glucose levels during an IpGTT at 4 months after transplantation (HOM CORR n = 6, HOM n = 6). (E) Plasma human C-peptide and (F) blood glucose levels during an IpGTT after 2-month dulaglutide treatment (HOM CORR n = 3, HOM n = 3). (D-F) Unpaired t-test with Holm-Śidák correction for multiple comparison. In time course line plots, data are shown as mean ± s.e.m..

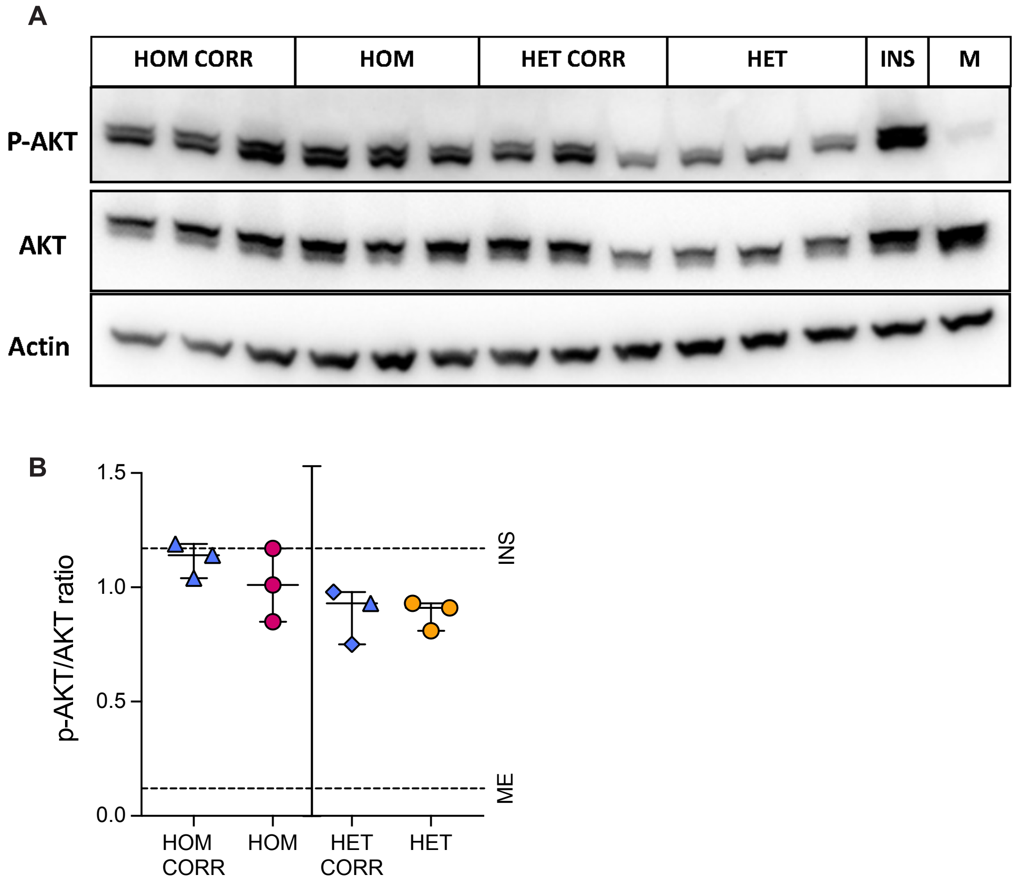

Appendix Figure S14. Normal signaling of insulin secreted from R6C mutant and isogenic corrected iPSC-islets

(A-B) HepG2 cells underwent 30-minute treatment with conditioned medium collected from homozygous (HOM, pink) and isogenic corrected (HOM CORR, blue), and heterozygous (HET, yellow) and isogenic corrected (HET CORR, blue) iPSC-islets. Negative control was media only (M) while positive control is media with 5 ng insulin (Novorapid, INS). HepG2 cells lysates (15 µg per sample) were separated on 4%–12% Bis-Tris gels, transferred to PVDF membranes, and immunoblotted for phosphorylated AKT (p-AKT, Protein Kinase B), total AKT, and β-Actin. (B) Quantification of p-AKT to total AKT ratio, n=3 independent experiments.

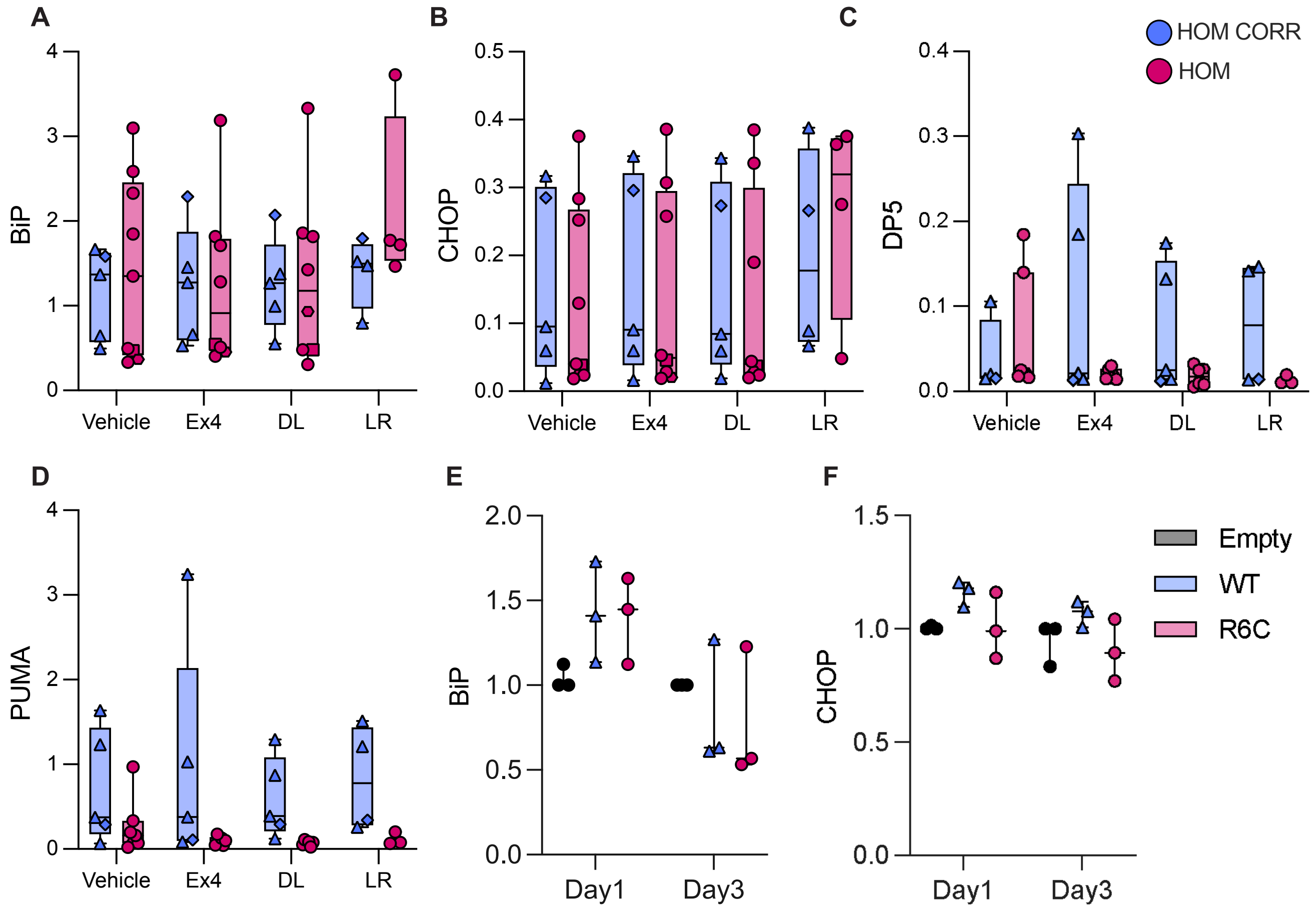

Appendix Figure S15. R6C *INS* does not induce ER stress

(A-D) *BiP*, *CHOP*, *DP5*, and *PUMA* mRNA expression of stage 7 iPSC-islets treated *in vitro* for 48 hours with glucagon-like peptide-1 receptor agonists exendin-4 (Ex4, 50 nM), dulaglutide (DL, 50 nM), and liraglutide (LR, 50 nM) or vehicle PBS. Sample sizes (n) for *BiP* and *CHOP* (HOM CORR vs. HOM) were: Vehicle (PBS) and Ex4 and DL, 5 vs. 9; LR, 4 vs. 4. Sample sizes (n) for *DP5* and *PUMA* (HOM CORR vs. HOM) were: Vehicle (PBS) and Ex4 and DL, 5 vs. 6; LR, 3 vs. 4. In box plots, the median of independent experiments is shown by a horizontal line; 25th and 75th percentiles are at the bottom and top of the boxes; whiskers represent minimum and maximum values. (E-F) EndoC-βH1 *INS*-knockout cells were transfected with plasmids expressing wildtype insulin (blue, WT), R6C insulin (pink, R6C), or only GFP (black, empty). *CHOP* and *BiP* mRNA expression in GFP-sorted cells was examined in n = 3 independent experiments. The mean is shown by a horizontal line; whiskers represent minimum and maximum values.

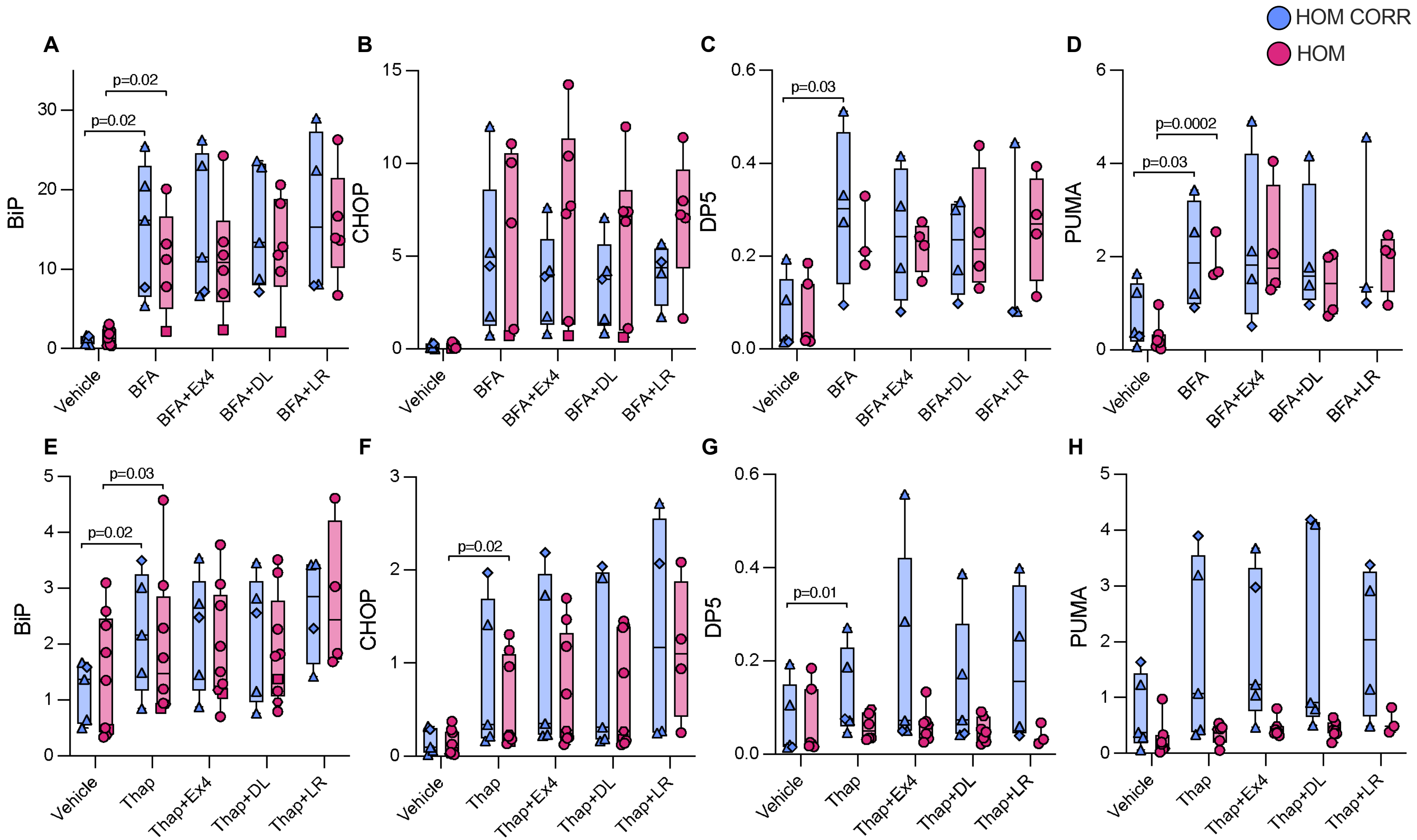

Appendix Figure S16. No worsening of ER stress in homozygous R6C β cells by ER stressors and GLP1-RAs

Homozygous (HOM, pink) and isogenic corrected (HOM CORR, blue) stage 7 iPSC-islets were treated with synthetic endoplasmic reticulum (ER) stressors (BFA: brefeldin A, 0.025 µg/mL, 24 hours or thapsigargin: Thap, 1 µM, 48 hours; Vehicle being DMSO) or in combination with glucagon-like peptide 1 receptor agonists (GLP-1RAs, 50 nM exendin-4: Ex4, dulaglutide: DL, or liraglutide: LR). mRNA expression of *BiP*, *CHOP*, *DP5* and *PUMA* by BFA (A-D) or Thap (E-H) in combination with GLP-1RAs (Ex4, DL, or LR). Mixed-effects analysis with Tukey correction for multiple comparisons. Sample sizes (n) for *BiP* and *CHOP* (HOM CORR vs. HOM) were: Vehicle (DMSO+PBS), 5 vs. 9; BFA, 5 vs. 9; BFA (+Ex4 and +DL), 5 vs. 6; BFA+LR, 4 vs. 5; Thap (and +Ex4 and +DL), 5 vs. 9; Thap+LR, 4 vs. 4. Sample sizes (n) for *DP5* and *PUMA* (HOM CORR vs. HOM) were: Vehicle (DMSO) and Thap (and +Ex4 and +DL), 5 vs. 7; BFA (and +Ex4 and +DL), 4 vs. 4; BFA/Thap+LR, 3 vs. 4. In box plots, the median of

independent experiments is shown by a horizontal line; 25th and 75th percentiles are at the bottom and top of the boxes; whiskers represent minimum and maximum values.

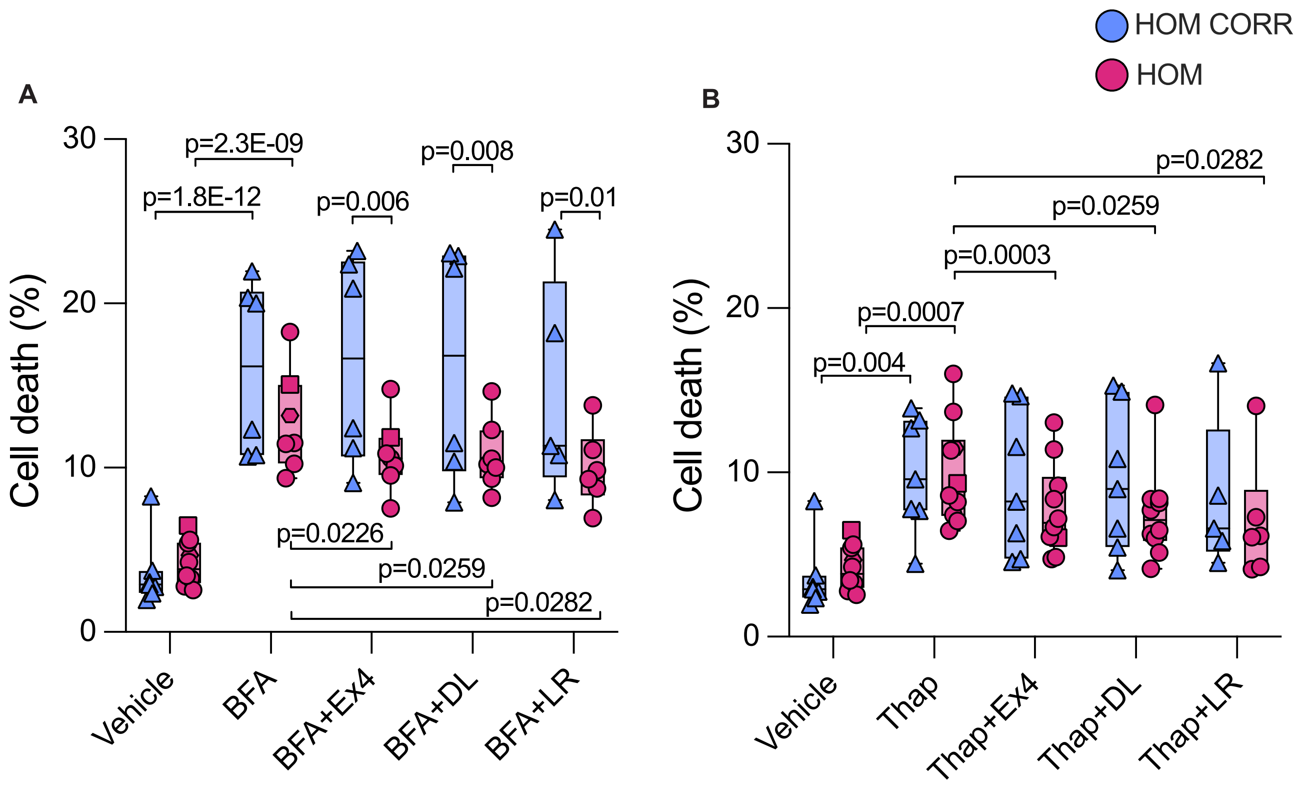

Appendix Figure S17. Protection against apoptosis in homozygous R6C β cells by GLP1-RAs

Homozygous (HOM, pink) and isogenic corrected (HOM CORR, blue) stage 7 iPSC-islets were treated with synthetic endoplasmic reticulum (ER) stressors (BFA: brefeldin A, 0.025 µg/mL, 24 hours or thapsigargin: Thap, 1 µM, 48 hours; Vehicle being DMSO) or in combination with glucagon-like peptide 1 receptor agonists (GLP-1RAs, 50 nM exendin-4: Ex4, dulaglutide: DL, or liraglutide: LR). Cell death (%) after exposure to BFA (A) or Thap (B) in combination with GLP-1RAs. Paired t-test comparing vehicle and Thap or BFA and mixed-effects analysis with Tukey correction for multiple comparisons between ER stressor and GLP-1RAs. Sample sizes for each treatment condition (HOM-CORR vs. HOM) were: Vehicle (DMSO+PBS), 7 vs. 10; BFA (and + Ex4 and + DL), 6 vs. 7; BFA + LR, 5 vs. 6. In box plots, the median of independent experiments is shown by a horizontal line; 25th and 75th percentiles are at the bottom and top of the boxes; whiskers represent minimum and maximum values.

Appendix tables and legend

Appendix Table S1. Allele frequency of *INS* R6C in population-level datasets

Dataset	Allele Frequency
TOPMed (freeze 8)	0.000030
All of Us	0.000018
Regeneron Genetics Center Million Exome (RGC-ME)	0.000026
gnomAD v4.1	0.000014
ALFA (Allele Frequency Aggregator)	0.000032
UK Biobank	0.000004
Geisinger Health System	0.000156

Appendix Table S2. *In silico* prediction of the *INS* R6C mutation

Predictor	Score	Interpretation	Threshold and Notes	Reference
REVEL	0.458	Likely benign	>0.5 damaging; <0.4 benign	(Ioannidis *et al*, 2016)
ClinPred	0.2543	Likely benign	>0.5 damaging; optimized for clinical variants	(Alirezaie *et al*, 2018)
AlphaMissense	0.1259, B	Likely benign	>0.5 damaging; B = likely benign	(Cheng *et al*, 2023)
MetaSVM	0.9525	Damaging	>0 damaging	(Kim *et al*, 2017)
CADD_phred	22.5	Damaging	≥20 top 1% most damaging	(Schubach *et al*, 2024)
DANN	0.9906	Damaging	>0.9 strongly damaging	(Quang *et al*, 2015)

Appendix Table S3. CRISPR/Cas9 editing and on-target and off-target profiling on pre- and post-CRISPR iPSCs

No.	Off-target Seq + PAM	mismatch position	mismatch number	mit score	cfd score	chromosome	start	end	strand
#	GGGCAGGAGGC**G**CATCCACA GGG	...........*........	1	49.2	0.72222	**chr11**	**2160945**	**2160967**	**+**
1	**T**G**A**CAGGAGGCACATCCAC**G** GGG	*.*................*	3	1.47120113	0.57353	**chr16**	**85261523**	**85261545**	**+**
2	**AT**G**A**AG**A**AGGCACATCCACA GGG	**.*..*.............	4	0.932255747	0.4823	**chr6**	**76721887**	**76721909**	**+**
3	GGG**A**AG**A**AGGCACATC**T**ACA TGG	...*..*.........*...	3	0.781869327	0.39298	**chr14**	**40521939**	**40521961**	**+**
4	G**CA**C**G**GGAGGCACAT**A**CACA GGG	.**.*..........*....	4	0.254924684	0.37385	**chr12**	**113062713**	**113062735**	**-**
5	G**A**GCAGGA**TT**CACATCCACA TGG	.*......**..........	3	1.503781857	0.36667	**chr3**	**119571998**	**119572020**	**+**
Off-targets	Mismatch								
No.	ULBi012.31	ULBi012.8	ULBi012.31 CR4	ULBi012.31 CR3	ULBi012.31 CR41	ULBi011.5	ULBi011.5 CR29	ULBi011.5 CR21	ULBi011.5 CR30
1	-	-	-	-	-	-	-	-	-
2	-	-	-	-	-	-	-	-	-
3	-	-	-	-	-	-	-	-	-
4	-	-	-	-	-	-	-	-	-
5	-	-	-	-	-	-	-	-	-

guideRNA + PAM (mutation red): GGGCAGGAGGC**A**CATCCACA GGG (On target score: 74.0, Off target score: 56.5)

Homology directed repair template:
ACCAGGTGTGAGCCGCACAGGTGTTGGTTCACAAAGGCTGCGGCTGGGTCAGGTCCCCAGAGGGCCAGCAGCGCCAGCAGTGGCAGGAGGCGCATCCACAGAGCCAT GGCAGAAGGACAGTGATCTGG

Designed CRISPR/Cas9 target on *INS*.

*: indicates mismatch, .: indicates match

All sequences are written from 5 prime to 3 prime

Appendix Table S4. ***In silico*** **prediction of structure and wild type and *INS* R6C signal peptide interaction with SRP54 and SEC61α2**

Metrics	SRP54		SEC61α2	
	WT	R6C	WT	R6C
ipTM*	0.75	0.55	0.42	0.45
pTM**	0.79	0.64	0.77	0.75
Buried surface area	1800.7	1790.5	1381.6	1236.1
Hydrogen bonds	8	1	0	0

*ipTM: interface predicted template modelling

**pTM: predicted template modelling

Appendix Table S5. Differentially expressed genes between corrected and homozygous R6C β cell-purified aggregates

ensembl_id	gene_symbol	baseMean	log2FoldChange	lfcSE	stat	pvalue	padj	change	mean TPM_COR	mean TPM_HOM
ENSG00000204065	TCEAL5	1920.16	6.83	0.49	13.92	4.53E-44	9.98E-40	Up	0.24	32.39
ENSG00000186094	AGBL4	276.71	6.07	0.48	12.72	4.44E-37	4.90E-33	Up	0.03	1.75
ENSG00000167981	ZNF597	418.66	12.00	1.06	11.32	1.05E-29	7.73E-26	Up	0.00	1.04
ENSG00000293469	ENSG00000293469	396.01	11.85	1.08	10.93	7.99E-28	4.40E-24	Up	0.00	1.30
ENSG00000197134	ZNF257	61.69	9.29	1.14	8.15	3.59E-16	1.58E-12	Up	0.00	0.60
ENSG00000108439	PNPO	3874.83	1.17	0.17	6.88	5.87E-12	2.16E-08	Up	6.31	14.18
ENSG00000261915	ENSG00000261915	48.95	-9.32	1.55	-6.00	1.92E-09	6.06E-06	Down	0.32	0.00
ENSG00000174028	FAM3C2P	79.94	19.69	3.38	5.82	5.88E-09	1.62E-05	Up	0.00	2.32
ENSG00000204789	ZNF204P	56.68	19.44	3.38	5.75	9.12E-09	2.24E-05	Up	0.00	0.52
ENSG00000267058	ENSG00000267058	130.23	2.38	0.45	5.28	1.28E-07	2.83E-04	Up	0.24	1.21
ENSG00000286449	ENSG00000286449	22.49	7.77	1.50	5.18	2.16E-07	4.34E-04	Up	0.00	0.08
ENSG00000198300	PEG3	4249.77	15.35	3.02	5.09	3.62E-07	6.65E-04	Up	0.00	6.55
ENSG00000224957	LINC01266	21.43	7.86	1.56	5.03	4.92E-07	8.35E-04	Up	0.00	0.08
ENSG00000166770	ZNF667-AS1	2517.56	14.70	3.01	4.88	1.04E-06	1.64E-03	Up	0.00	25.85
ENSG00000164932	CTHRC1	351.79	1.96	0.40	4.86	1.18E-06	1.73E-03	Up	1.09	3.79
ENSG00000155974	GRIP1	776.93	1.63	0.34	4.79	1.68E-06	2.31E-03	Up	0.86	2.85
ENSG00000022556	NLRP2	1619.23	14.12	3.03	4.66	3.18E-06	4.12E-03	Up	0.00	5.93

ENSG00000204179	PTPN20	32.40	8.26	1.78	4.64	3.43E-06	4.21E-03	Up	0.00	0.36
ENSG00000132481	TRIM47	37.08	-2.99	0.66	-4.51	6.40E-06	7.43E-03	Down	0.38	0.06
ENSG00000159915	ZNF233	75.93	4.42	1.00	4.44	9.04E-06	9.97E-03	Up	0.02	0.45
ENSG00000290263	ENSG00000290263	25.85	8.02	1.81	4.43	9.62E-06	1.01E-02	Up	0.00	0.06
ENSG00000198046	ZNF667	498.19	12.29	2.83	4.34	1.41E-05	1.41E-02	Up	0.00	1.68
ENSG00000188385	JAKMIP3	84.62	-2.79	0.65	-4.32	1.54E-05	1.48E-02	Down	0.30	0.09
ENSG00000239572	ENSG00000239572	64.18	2.12	0.50	4.19	2.77E-05	2.55E-02	Up	0.06	0.26
ENSG00000113209	PCDHB5	50.92	3.86	0.94	4.10	4.09E-05	3.61E-02	Up	0.02	0.26
ENSG00000095303	PTGS1	61.44	-4.26	1.04	-4.07	4.61E-05	3.91E-02	Down	0.33	0.01
ENSG00000272674	PCDHB16	4572.04	1.23	0.30	4.06	4.87E-05	3.98E-02	Up	5.88	13.62
ENSG00000198355	PIM3	3656.60	-1.28	0.32	-4.03	5.51E-05	4.34E-02	Down	22.42	11.68
ENSG00000105877	DNAH11	41.60	-4.27	1.07	-4.01	6.11E-05	4.40E-02	Down	0.07	0.00
ENSG00000151687	ANKAR	123.98	-1.60	0.40	-4.00	6.29E-05	4.40E-02	Down	0.99	0.46
ENSG00000167785	ZNF558	225.48	11.26	2.81	4.01	6.16E-05	4.40E-02	Up	0.00	1.21
ENSG00000248406	ENSG00000248406	21.99	7.96	1.99	3.99	6.48E-05	4.40E-02	Up	0.00	0.18
ENSG00000257228	ENSG00000257228	376.20	2.22	0.56	3.99	6.58E-05	4.40E-02	Up	1.44	6.13
ENSG00000183889	NPIPA6	573.71	-1.90	0.48	-3.95	7.93E-05	5.00E-02	Down	2.73	5.83
ENSG00000226816	ENSG00000226816	631.39	3.20	0.81	3.95	7.85E-05	5.00E-02	Up	0.47	4.14

Appendix Table S6. Custom curated gene-wise z-scores of key β cell function pathways

category	genes	mean_z_CORR	mean_z_HOM
Beta cell identity	*INS, PDX1, NKX6-1, MAFA, NEUROD1, NKX2-2, PAX6*	-0.39	0.31
Cytosolic stress	*HSPA1A, HSPA1B, HSPA6, DNAJB1, HSP90AA1, HSPB1, BAG3*	0.10	-0.08
ER translocation	*SRP54, SEC61A2, TRAM1, SSR1, SSR3, SEC62, SEC63*	-0.27	0.21
ER-Golgi transportation	*SEC23B, SEC24B, COPB1, SEC16A, MIA3, SURF4, LMAN1*	-0.33	0.26
ERAD and pEQC	*SYVN1, SEL1L, DERL1, VCP, HERPUD1, RNF149, BAG6*	0.09	-0.07
Insulin processing	*P4HB, PCSK1, PCSK2, CPE, ERO1A, PDIA6, HSP90B1*	-0.07	0.06
Insulin secretion	*GCK, SLC2A2, G6PC2, KCNJ11, CACNA1D, HNF1B, PTPRN*	0.03	-0.02
UPR	*DDIT3, ATF4, HSPA5, DNAJC3, EIF2AK3, ERN1, WFS1*	-0.01	0.01

ER: endoplasmic reticulum
ERAD: ER-associated degradation
pEQC: preemptive quality control
UPR: unfolded protein response
HOM: *INS* R6C homozygous
CORR: *INS* R6C homozygous corrected

Appendix Table S7. qPCR primers

Sendai Virus transgene	AGACCCTAAGAGGACGAAGACAGA	Forward
	ACTCCCATGGCGTAACTCCATAG	Reverse
Human ACTB	CTGTACGCCAACACAGTGCT	Forward
	GCTCAGGAGGAGCAATGATC	Reverse
Human VAPA	TACCGAAACAAGGAAACTAATGGAA	Forward
	GCCTTAAACCTTCATCTCTCAGGT	Reverse
Human TFRC	CCACTGAATGGCTAGAGGGATA	Forward
	GCTGGCAGAAACCTTGAAGTTG	Reverse
Human INS	CCAGCCGCAGCCTTTGTGA	Forward
	CCAGCTCCACCTGCCCCA	Reverse
Human GCG	GCTAAACAGAGCTGGAGAGTAT	Forward
	AAGCCCTCTTTGGGAACTT	Reverse
Human SST	GTTTGACCAGCCACTCTCCAG	Forward
	TACTTGGCCAGTTCCTGCTTCC	Reverse
Human NKX6.1	GGGCTCGTTTGGCCTATT	Forward
	CGTGCTTCTTCCTCCACTT	Reverse
Human PDX1	AAAGCTCACGCGTGGAAA	Forward
	GCCGTGAGATGTACTTGTTGA	Reverse
Human SOX9	ATCAAGACGGAGCAGCTGAG	Forward
	GGCTGTAGTGTGGGAGGTTG	Reverse
Human NGN3	GACGACGCGAAGCTCACCAA	Forward
	TACAAGCTGTGGTCCGCTAT	Reverse
Human NKX2.2	GAACCCCTTCTACGACAGCA	Forward
	ACCGTGCAGGGAGTACTGAA	Reverse
Human NEUROD1	CTATCACTGCTCAGGACCTACT	Forward
	CCACTCTCGCTGTACGATTT	Reverse
Human GLP-1R	AAGGACAACTCCAGCCTGC	Forward
	ATGATGTAGAGGAACAGGAG	Reverse
Human CHOP	Hs_DDIT3_1_SG Quantitect Primer Assay (Qiagen)	Forward & Reverse
Human BiP	Hs_HSPA5_1_SG QuantiTect Primer Assay (Qiagen)	Forward & Reverse
Human DP5	GAGCCCAGAGCTTGAAAGG	Forward
	CCCAGTCCCATTCTGTGTTT	Reverse
Human PUMA	TTGTGCTGGTGCCCGTTCCA	Forward

	AGGCTAGTGGTCACGTTTGGCT	Reverse
Human CHOP (EndoC-βH1)	GCTACTGACTACCCTCTCACTA	Forward
	TACAAGCTGAGACCTTTCCTTT	Reverse
Human BiP (EndoC-βH1)	CCTTCGATGTGTCTCTTCTCAC	Forward
	ACGCTGGTCAAAGTCTTCTC	Reverse
Human Cyclo-A (EndoC-βH1)	GCCGAGGAAAACCGTGTACT	Forward
	TGCTGTCTTTGGGACCTTGTGT	Reverse
Human INS Sanger	TCTGCGGTCATCAAATGAGGG	Forward
	GCCATCAAGCAGGTCTGTTCC	Reverse
Human CRIPSR off-target 1	GCCCTAACCATACCCAGTGTC	Forward
	CGTGGGATGATCTGTCCGAAA	Reverse
Human CRIPSR off-target 2	GCATTCCAGGGAGAGTAAATGT	Forward
	TCTCAGCAAGAGCCTTCTCAC	Reverse
Human CRIPSR off-target 3	ATTTGGTGTGATTTGAGGTGGA	Forward
	GAGGAAAGAGATGGGATACATTGG	Reverse
Human CRIPSR off-target 4	TTCTGAACCTGAGCATGGGGTAA	Forward
	GGACACCACCGTGATTGATCT	Reverse
Human CRIPSR off-target 5	ATTGTAAACCCTGGAAGCGGAA	Forward
	TCTAACAAGCAGGCAGGAATCAA	Reverse

Appendix Table S8. Antibodies

Antibody	Company, Cat#, RRID	Dilution
Rabbit anti-human OCT4	Cell Signaling Technology Cat# 2840, RRID:AB_2167691	1 to 400
Mouse anti-human TRA1-60	Thermofisher Scientific, Cat#MA1-023, RRID: AB_2536699	1 to 100
Mouse anti-human SSEA4	Thermofisher Scientific, Cat#MA1-021, RRID: AB_2536687	1 to 500
Rabbit anti-human Nanog	Cell Signalling, Cat#4903, RRID:10559205	1 to 400
Goat anti-human SOX17	R and D Systems Cat# AF1924, RRID:AB_355060	1 to 500
Rabbit anti-human vimentin	Abcam Cat#137321, RRID:NA	1 to 500
Mouse anti-human beta tubulin III	Promega Cat#G7121, RRID:AB_430874	1 to 500
Mouse anti-human NKX6.1	BD Biosciences Cat# 563022, RRID:AB_2737958	1 to 250
Goat anti-human PDX1	R and D Systems Cat# AF2419, RRID:AB_355257	1 to 500
Guinea pig anti-human INS	Dako Cat# A0564; RRID:AB_10013624	1 to 2
Mouse anti-human GCG	Sigma-Aldrich Cat# G2654; RRID:AB_259852	1 to 1000
Rabbit anti-human SST	Abcam Cat#ab108456; RRID: AB_11158517	1 to 1000
Anti-CD49a antibody PE conjugated	BD Bioscience Cat#559596; RIDD: AB_397288	20 µL per 1E6 cells

Anti-PE microbeads	Miltenyi Cat#130-105-639; RIDD: NA	20 μL per 1E7 cells
Mouse anti-human proinsulin B-C junction sequence KTRREAEDLQ	Abmart, Cat# B-C junction; RRID: AB_2921300	1 to 1000
Mouse anti-human β-Actin (HepG2)	Proteintech, Cat# CL555-66009, RRID: AB_2919667	1 to 1000
Rabbit anti-human p-AKT (HepG2)	Cell Signaling Technology,4060S; RRID: AB_2315049	1 to 1000
Rabbit anti-human AKT (HepG2)	Cell Signaling Technology,4685S; RRID: AB_2225340	1 to 1000
anti-human β-Actin (HepG2)	Sigma, Cat#A5441, RRID: AB_476744	1 to 1000
