# Supplementary figures and images for: A new form of diabetes caused by INS mutations defined by zygosity, stem cell and population data
Source: EMBO Mol Med. 2026 Jan 3;18(2):620–45. doi: 10.1038/s44321-025-00362-9 (PMC12905373; doi:10.1038/s44321-025-00362-9)

## Slide 1
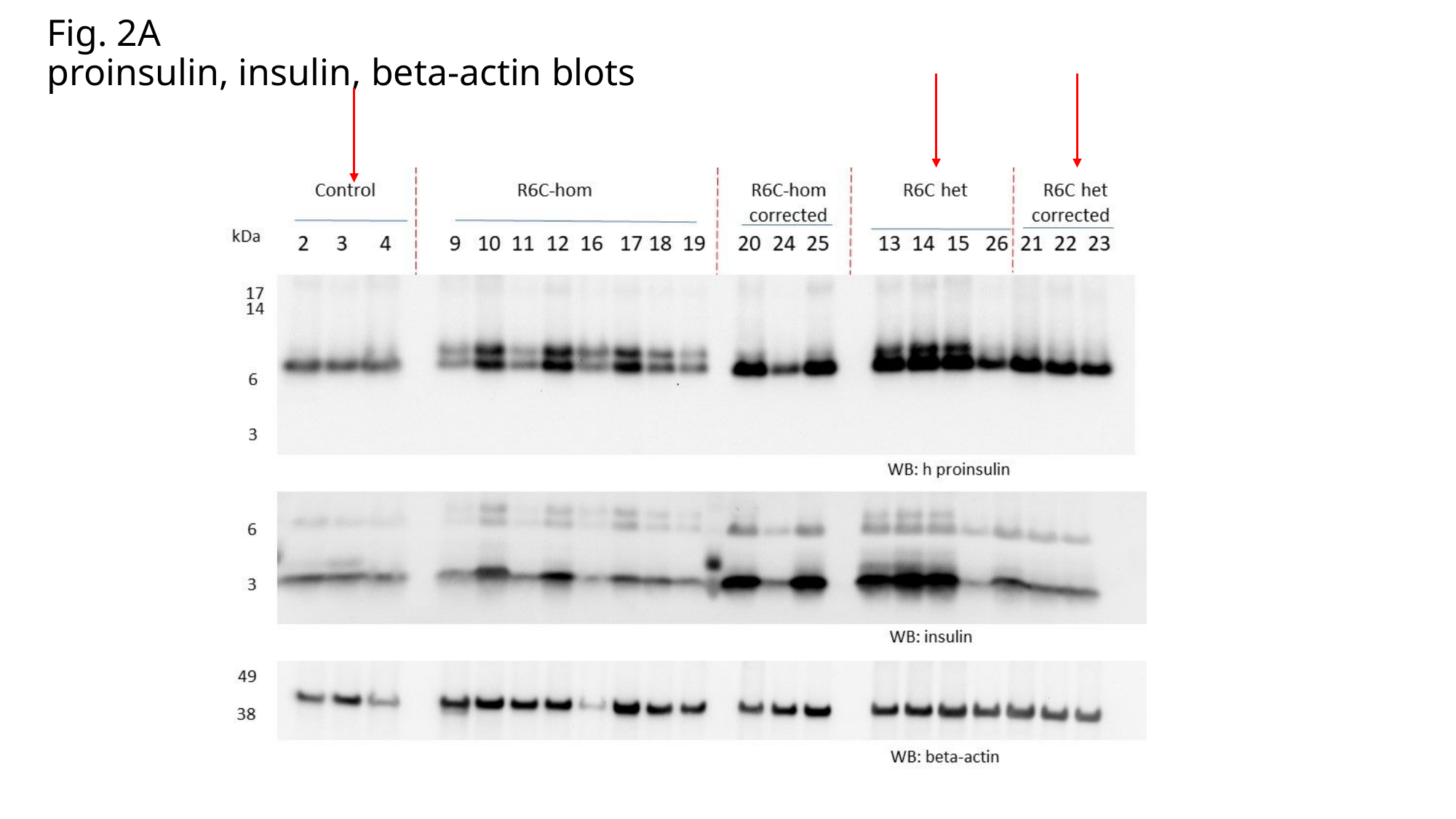

Fig. 2A
proinsulin, insulin, beta-actin blots

## Slide 2
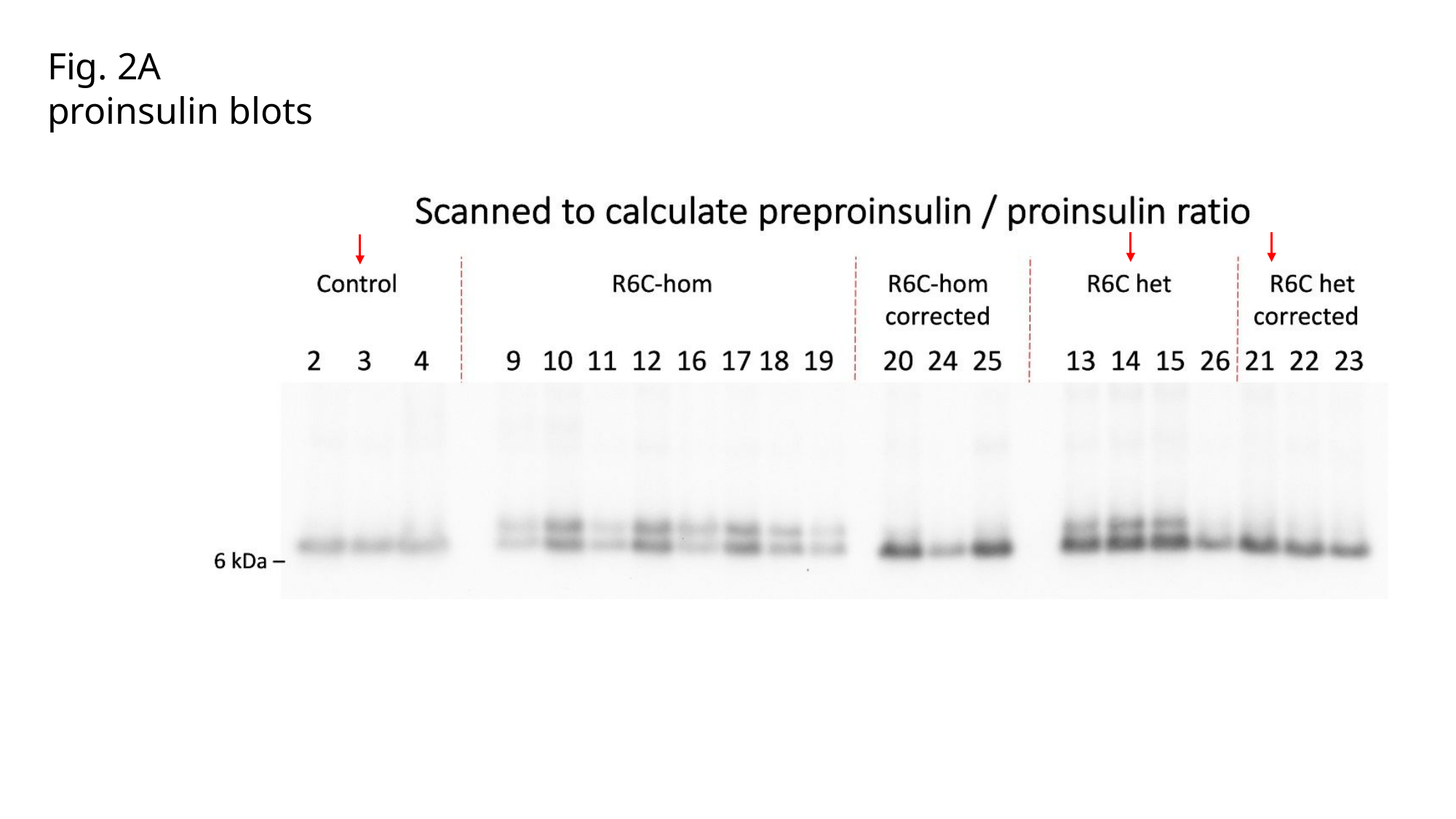

# Fig. 2A proinsulin blots

Supplement: Supplementary file 4 — Source data Fig. 2 [file 44321_2025_362_MOESM4_ESM.zip › Figure 2/2A/2A.pptx]

## Slide 1
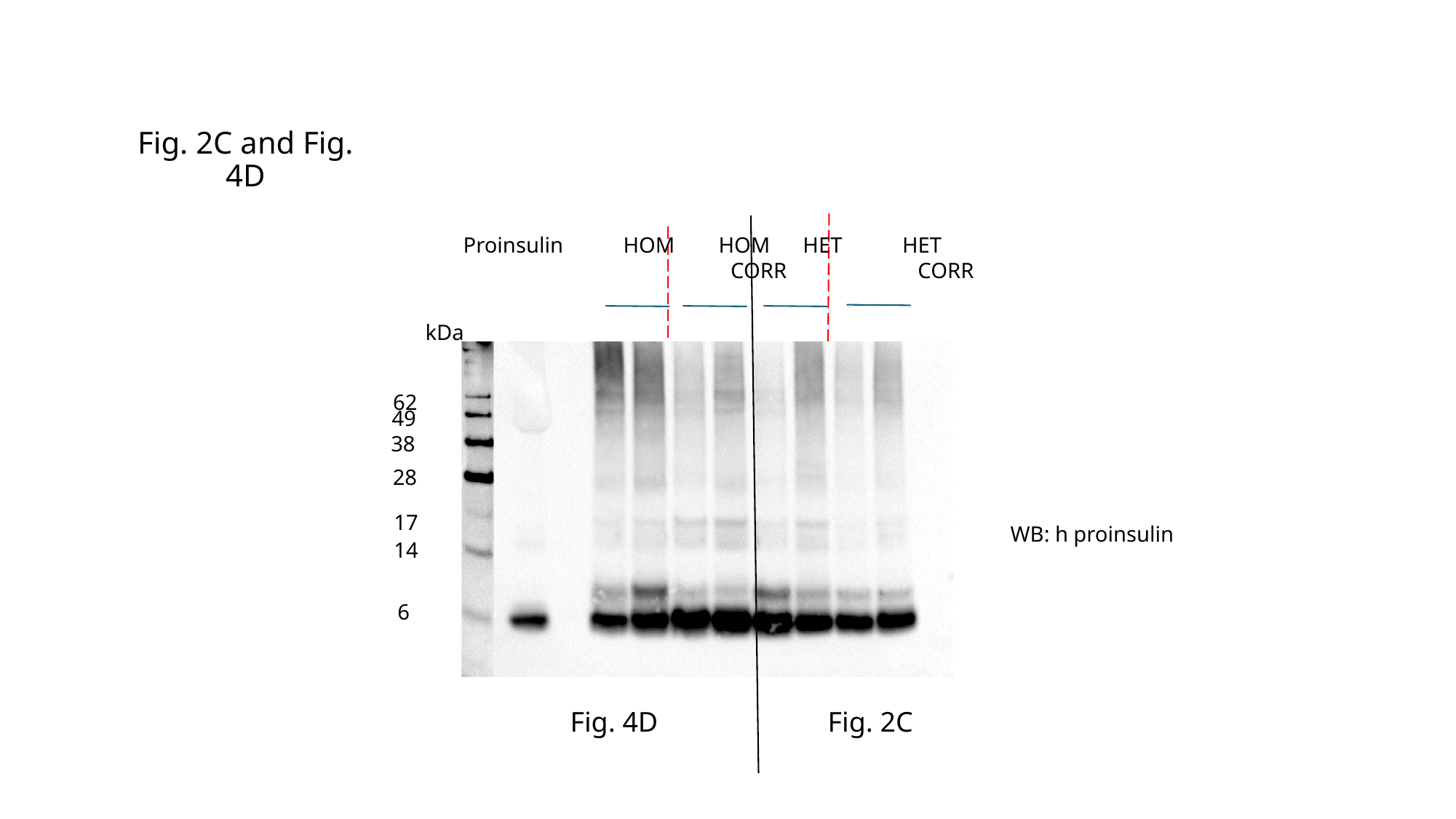

# Fig. 2C and Fig. 4D
 Proinsulin HOM HOM HET HET
 CORR CORR
kDa
62
49
38
28
17
WB: h proinsulin
14
6
Fig. 4D Fig. 2C

Supplement: Supplementary file 4 — Source data Fig. 2 [file 44321_2025_362_MOESM4_ESM.zip › Figure 2/2C/2C.pptx]

## Slide 1
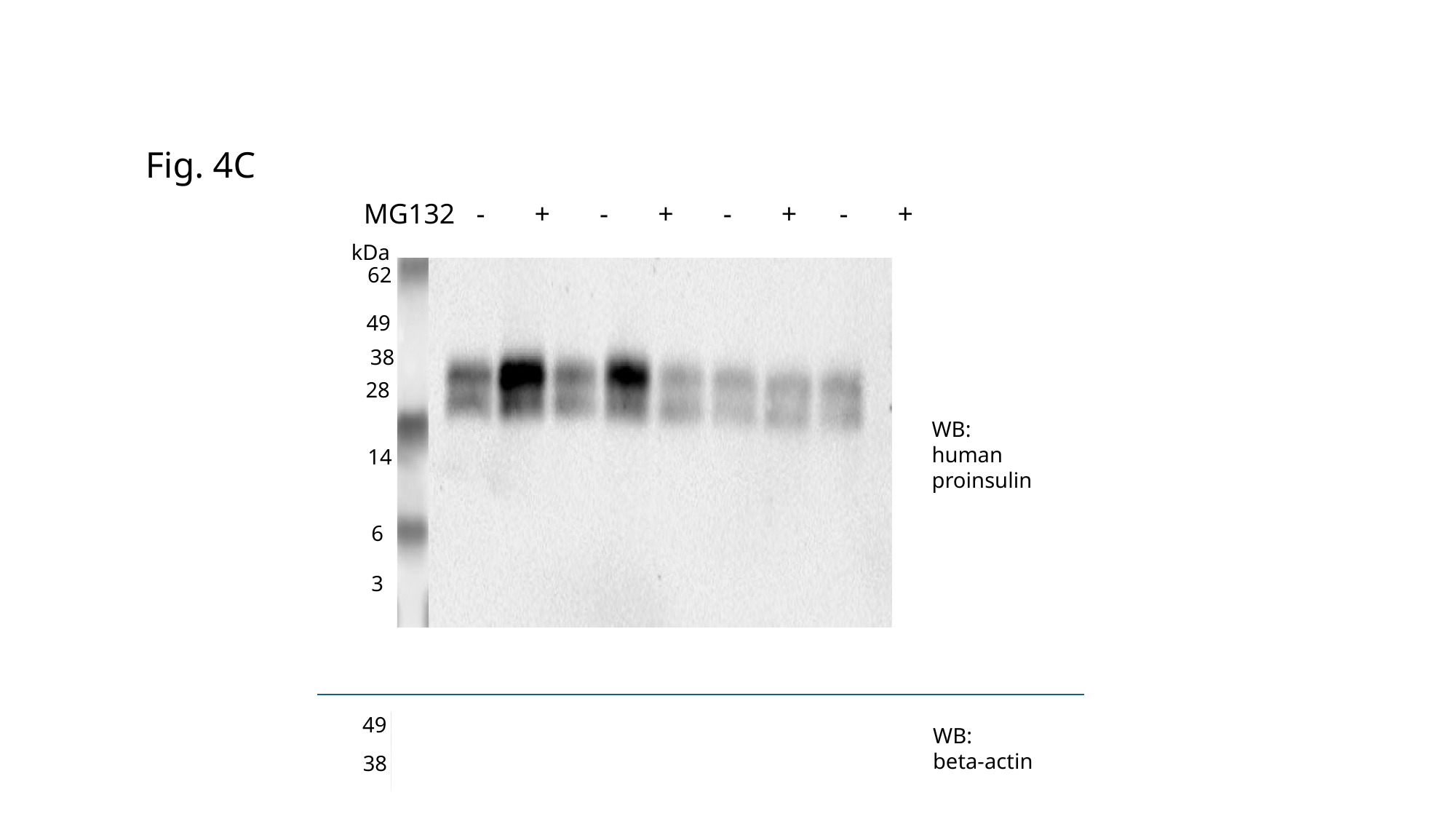

# Fig. 4C
MG132 - + - + - + - +
kDa
62
49
38
28
WB: human
proinsulin
14
6
3
49
WB:
beta-actin
38

Supplement: Supplementary file 6 — Source data Fig. 4 [file 44321_2025_362_MOESM6_ESM.zip › Figure 4/4C/4C.pptx]

## Slide 1
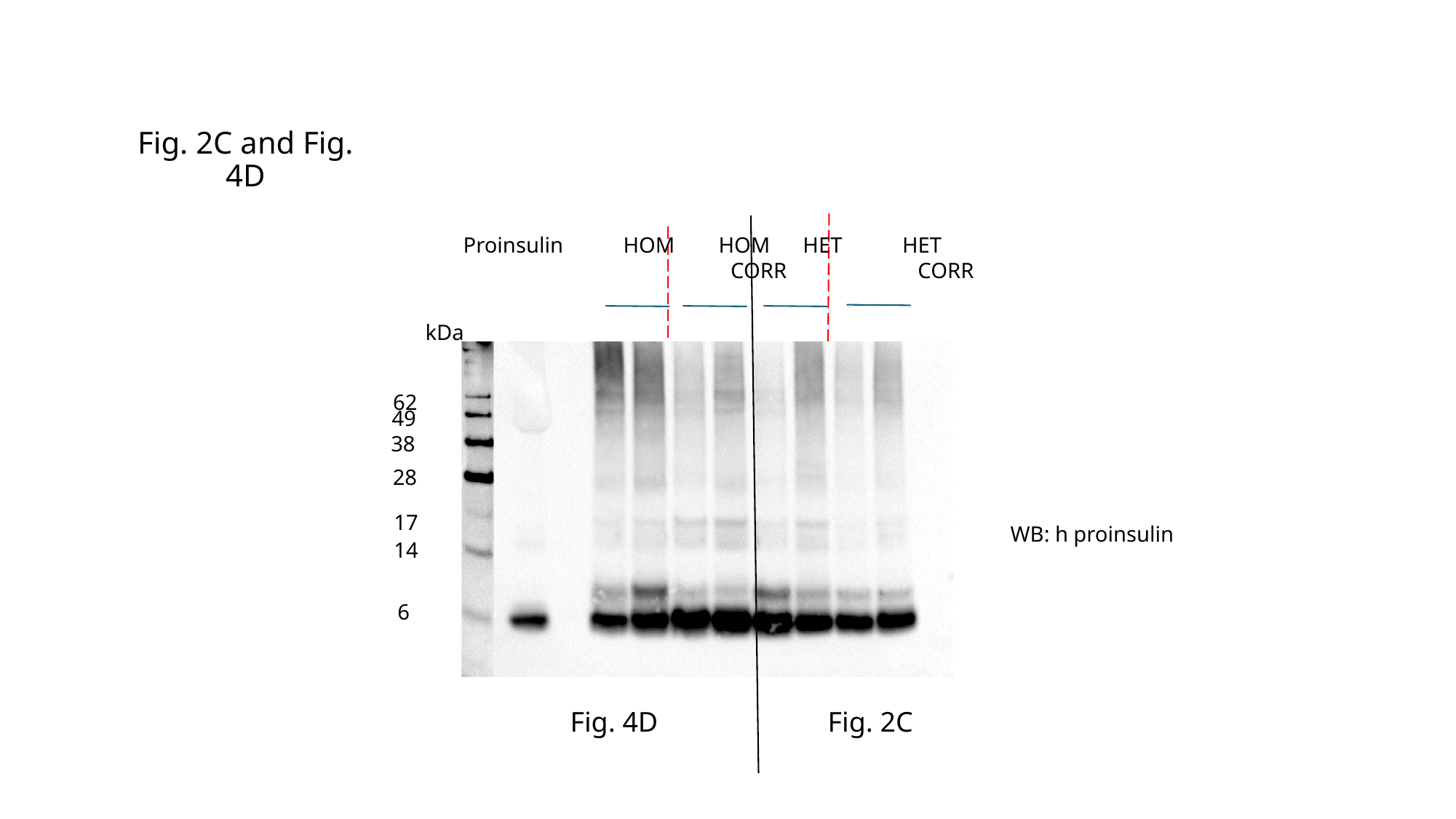

# Fig. 2C and Fig. 4D
 Proinsulin HOM HOM HET HET
 CORR CORR
kDa
62
49
38
28
17
WB: h proinsulin
14
6
Fig. 4D Fig. 2C

Supplement: Supplementary file 6 — Source data Fig. 4 [file 44321_2025_362_MOESM6_ESM.zip › Figure 4/4D/4D.pptx]

## Slide 1
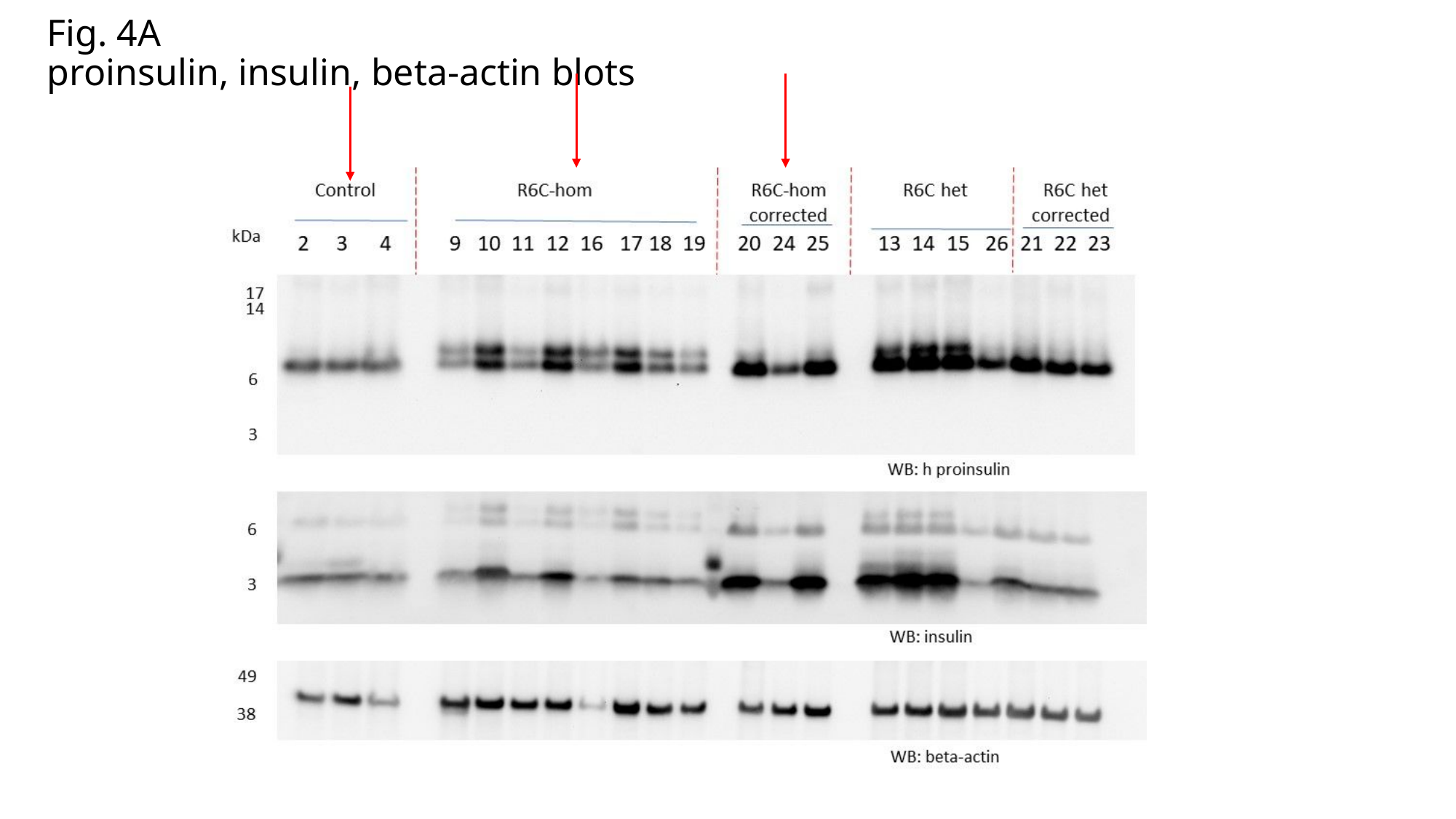

Fig. 4A
proinsulin, insulin, beta-actin blots

## Slide 2
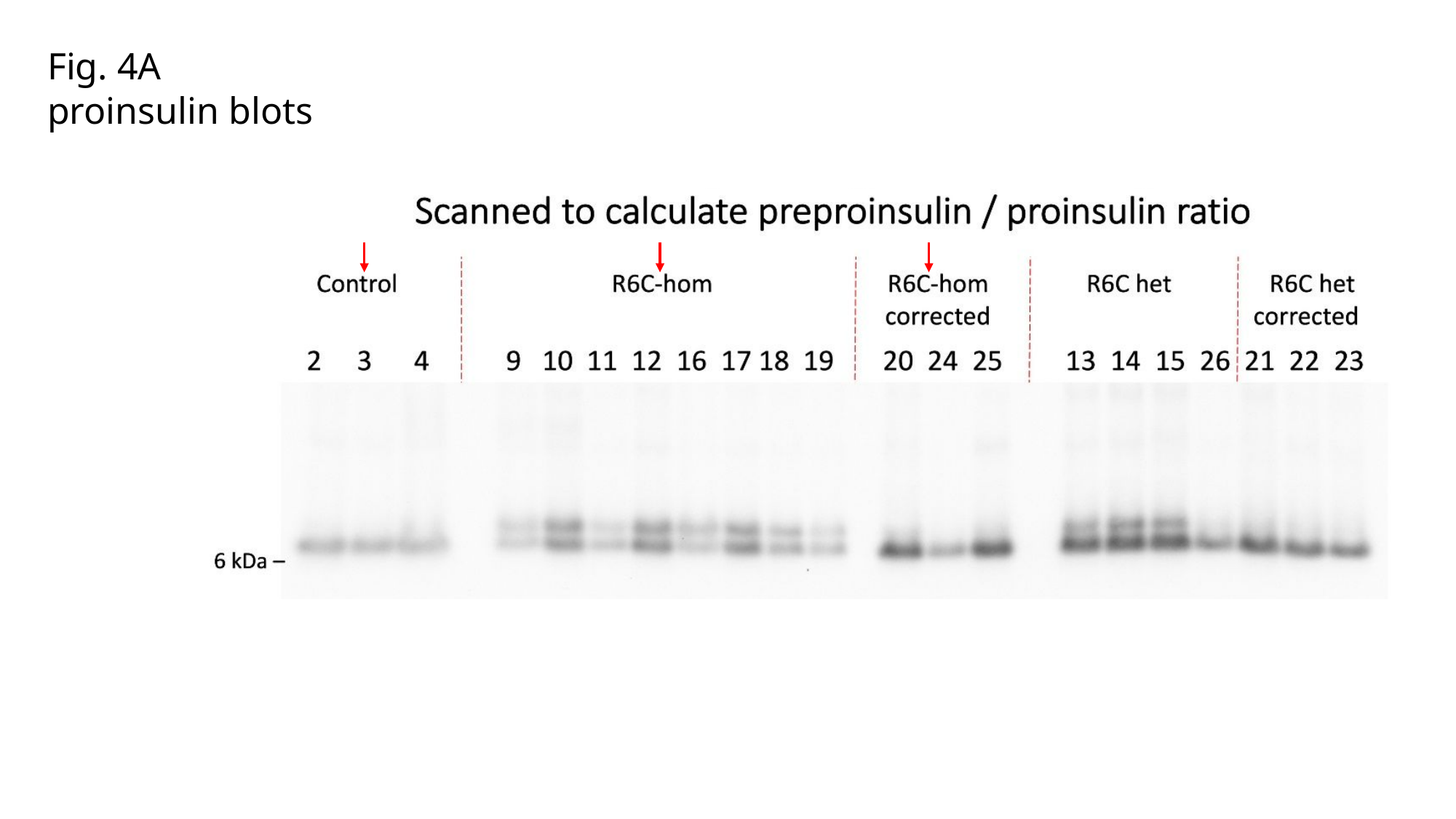

# Fig. 4A proinsulin blots

Supplement: Supplementary file 6 — Source data Fig. 4 [file 44321_2025_362_MOESM6_ESM.zip › Figure 4/4A/4A.pptx]

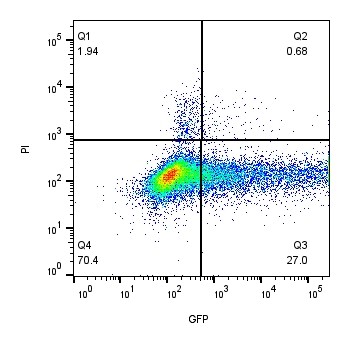

Supplement: Supplementary file 12 — Appendix Figure Source Data [file 44321_2025_362_MOESM12_ESM.zip › S2-facs-dot-plots/No.1/WT-D3.jpg]

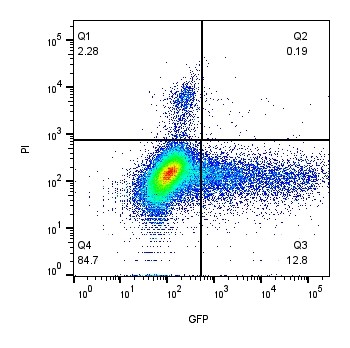

Supplement: Supplementary file 12 — Appendix Figure Source Data [file 44321_2025_362_MOESM12_ESM.zip › S2-facs-dot-plots/No.1/WT-D1.jpg]

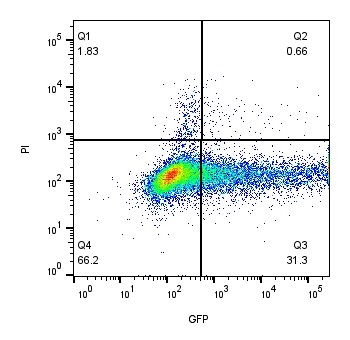

Supplement: Supplementary file 12 — Appendix Figure Source Data [file 44321_2025_362_MOESM12_ESM.zip › S2-facs-dot-plots/No.1/W+R-D3.jpg]

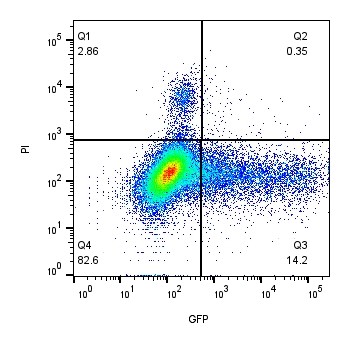

Supplement: Supplementary file 12 — Appendix Figure Source Data [file 44321_2025_362_MOESM12_ESM.zip › S2-facs-dot-plots/No.1/W+R-D1.jpg]

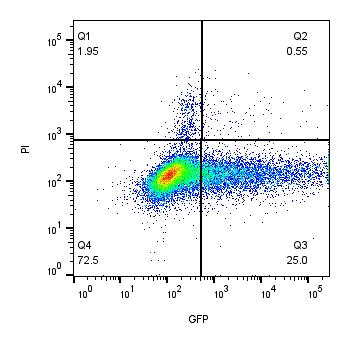

Supplement: Supplementary file 12 — Appendix Figure Source Data [file 44321_2025_362_MOESM12_ESM.zip › S2-facs-dot-plots/No.1/R-D3.jpg]

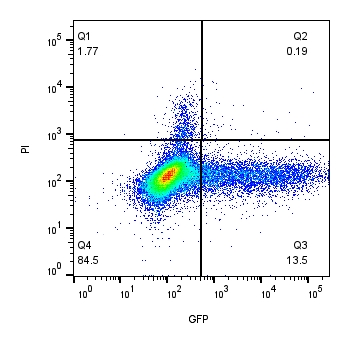

Supplement: Supplementary file 12 — Appendix Figure Source Data [file 44321_2025_362_MOESM12_ESM.zip › S2-facs-dot-plots/No.1/empty-D3.jpg]

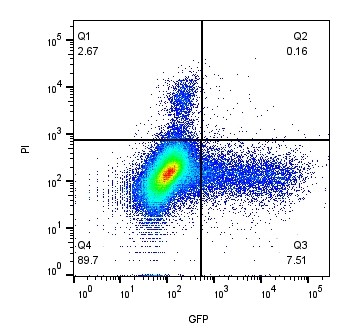

Supplement: Supplementary file 12 — Appendix Figure Source Data [file 44321_2025_362_MOESM12_ESM.zip › S2-facs-dot-plots/No.1/empty-D1.jpg]

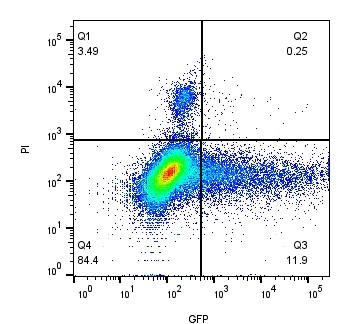

Supplement: Supplementary file 12 — Appendix Figure Source Data [file 44321_2025_362_MOESM12_ESM.zip › S2-facs-dot-plots/No.1/R-D1.jpg]

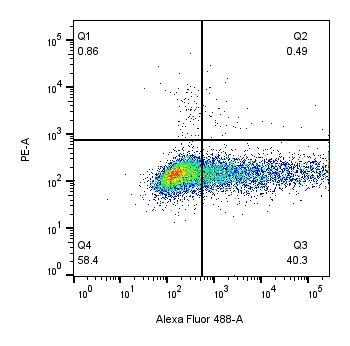

Supplement: Supplementary file 12 — Appendix Figure Source Data [file 44321_2025_362_MOESM12_ESM.zip › S2-facs-dot-plots/No.3/DAY3-WR1.jpg]

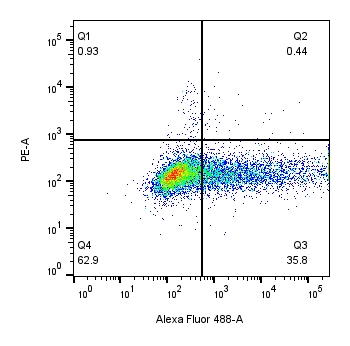

Supplement: Supplementary file 12 — Appendix Figure Source Data [file 44321_2025_362_MOESM12_ESM.zip › S2-facs-dot-plots/No.3/DAY3-R1.jpg]

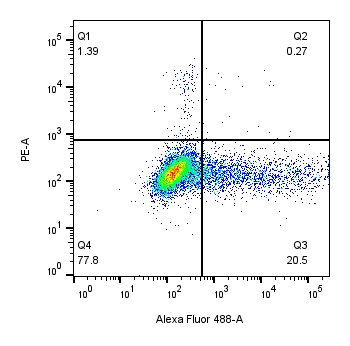

Supplement: Supplementary file 12 — Appendix Figure Source Data [file 44321_2025_362_MOESM12_ESM.zip › S2-facs-dot-plots/No.3/DAY1-WR1.jpg]

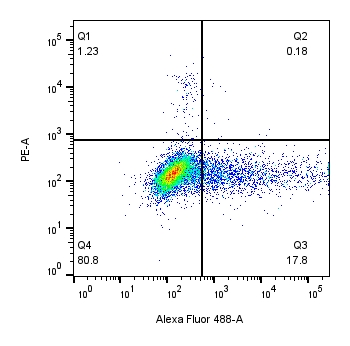

Supplement: Supplementary file 12 — Appendix Figure Source Data [file 44321_2025_362_MOESM12_ESM.zip › S2-facs-dot-plots/No.3/DAY1-R1.jpg]

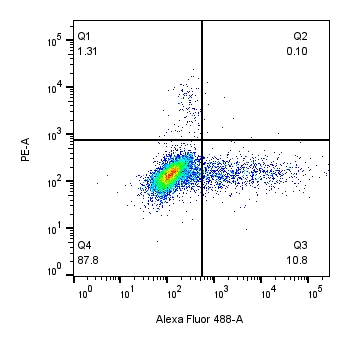

Supplement: Supplementary file 12 — Appendix Figure Source Data [file 44321_2025_362_MOESM12_ESM.zip › S2-facs-dot-plots/No.3/DAY3-E1.jpg]

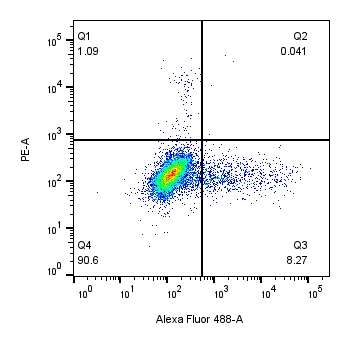

Supplement: Supplementary file 12 — Appendix Figure Source Data [file 44321_2025_362_MOESM12_ESM.zip › S2-facs-dot-plots/No.3/DAY1-E1.jpg]

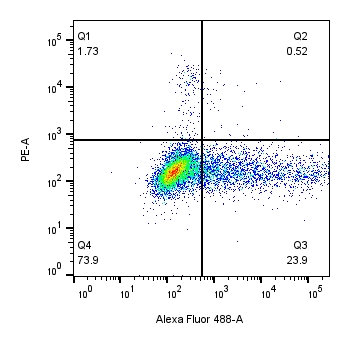

Supplement: Supplementary file 12 — Appendix Figure Source Data [file 44321_2025_362_MOESM12_ESM.zip › S2-facs-dot-plots/No.3/DAY1-W1.jpg]

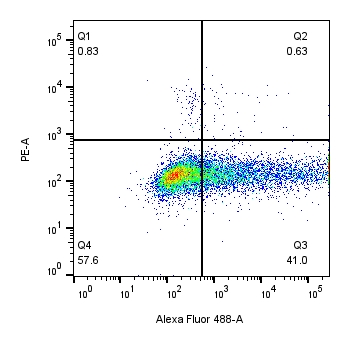

Supplement: Supplementary file 12 — Appendix Figure Source Data [file 44321_2025_362_MOESM12_ESM.zip › S2-facs-dot-plots/No.3/DAY3-W1.jpg]

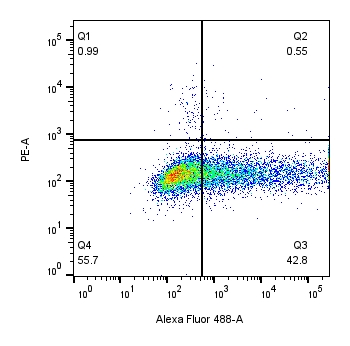

Supplement: Supplementary file 12 — Appendix Figure Source Data [file 44321_2025_362_MOESM12_ESM.zip › S2-facs-dot-plots/No.4/DAY3-WR2.jpg]

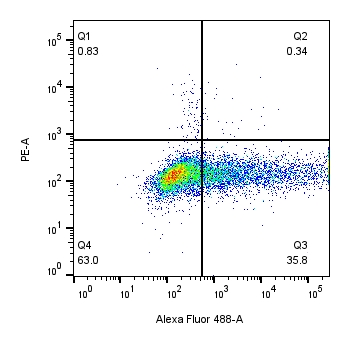

Supplement: Supplementary file 12 — Appendix Figure Source Data [file 44321_2025_362_MOESM12_ESM.zip › S2-facs-dot-plots/No.4/DAY3-R2.jpg]

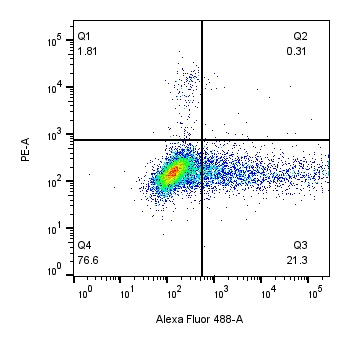

Supplement: Supplementary file 12 — Appendix Figure Source Data [file 44321_2025_362_MOESM12_ESM.zip › S2-facs-dot-plots/No.4/DAY1-WR2.jpg]

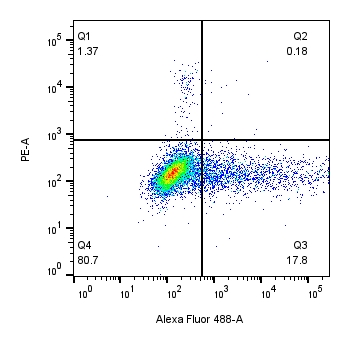

Supplement: Supplementary file 12 — Appendix Figure Source Data [file 44321_2025_362_MOESM12_ESM.zip › S2-facs-dot-plots/No.4/DAY1-R2.jpg]

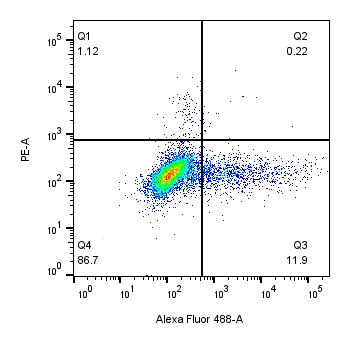

Supplement: Supplementary file 12 — Appendix Figure Source Data [file 44321_2025_362_MOESM12_ESM.zip › S2-facs-dot-plots/No.4/DAY3-E2.jpg]

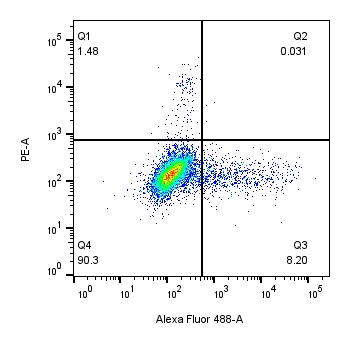

Supplement: Supplementary file 12 — Appendix Figure Source Data [file 44321_2025_362_MOESM12_ESM.zip › S2-facs-dot-plots/No.4/DAY1-E2.jpg]

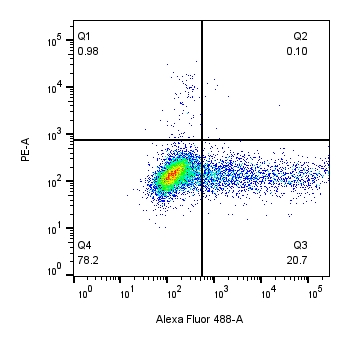

Supplement: Supplementary file 12 — Appendix Figure Source Data [file 44321_2025_362_MOESM12_ESM.zip › S2-facs-dot-plots/No.4/DAY1-W2.jpg]

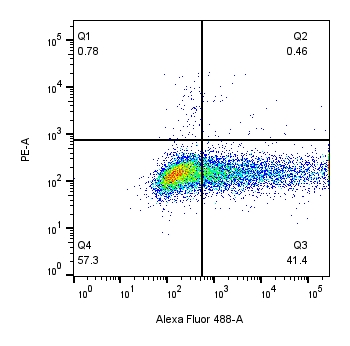

Supplement: Supplementary file 12 — Appendix Figure Source Data [file 44321_2025_362_MOESM12_ESM.zip › S2-facs-dot-plots/No.4/DAY3-W2.jpg]

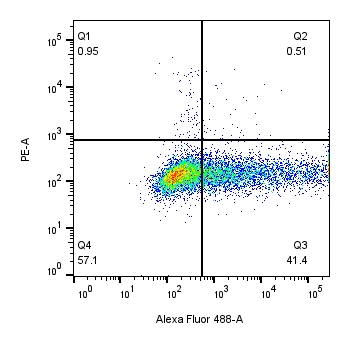

Supplement: Supplementary file 12 — Appendix Figure Source Data [file 44321_2025_362_MOESM12_ESM.zip › S2-facs-dot-plots/No.5/DAY3-WR3.jpg]

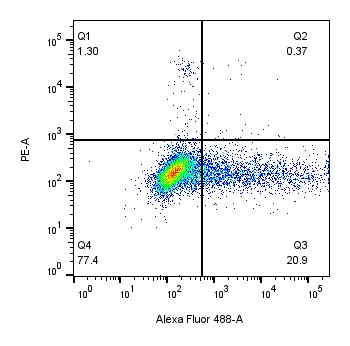

Supplement: Supplementary file 12 — Appendix Figure Source Data [file 44321_2025_362_MOESM12_ESM.zip › S2-facs-dot-plots/No.5/DAY1-WR3.jpg]

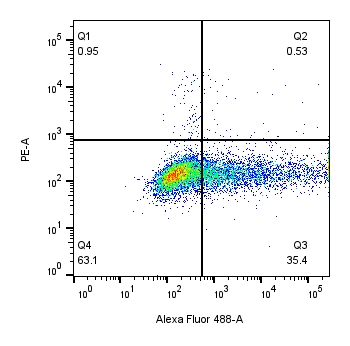

Supplement: Supplementary file 12 — Appendix Figure Source Data [file 44321_2025_362_MOESM12_ESM.zip › S2-facs-dot-plots/No.5/DAY3-R3.jpg]

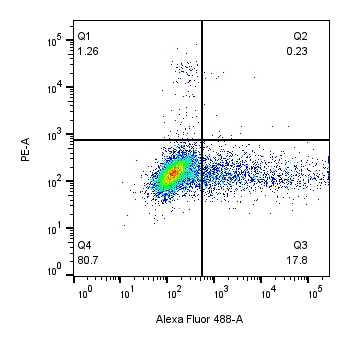

Supplement: Supplementary file 12 — Appendix Figure Source Data [file 44321_2025_362_MOESM12_ESM.zip › S2-facs-dot-plots/No.5/DAY1-R3.jpg]

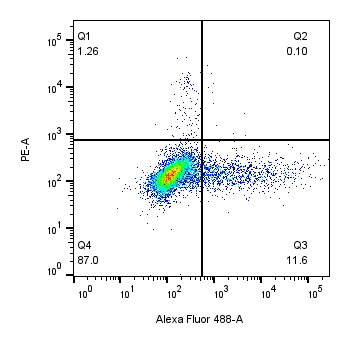

Supplement: Supplementary file 12 — Appendix Figure Source Data [file 44321_2025_362_MOESM12_ESM.zip › S2-facs-dot-plots/No.5/DAY3-E3.jpg]

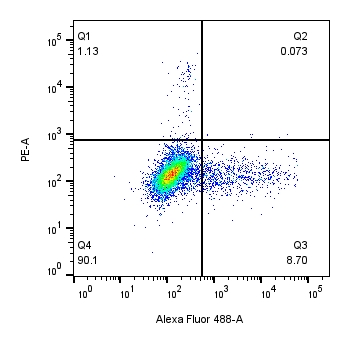

Supplement: Supplementary file 12 — Appendix Figure Source Data [file 44321_2025_362_MOESM12_ESM.zip › S2-facs-dot-plots/No.5/DAY1-E3.jpg]

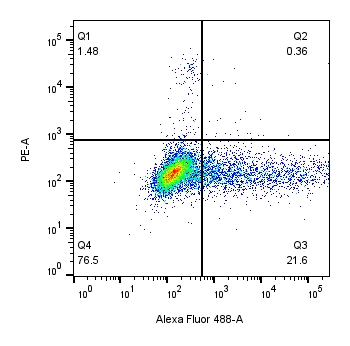

Supplement: Supplementary file 12 — Appendix Figure Source Data [file 44321_2025_362_MOESM12_ESM.zip › S2-facs-dot-plots/No.5/DAY1-W3.jpg]

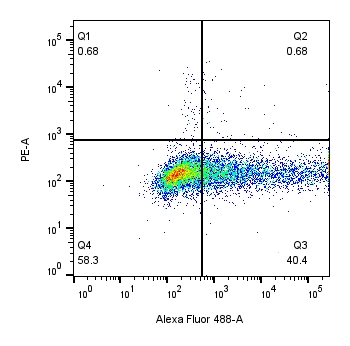

Supplement: Supplementary file 12 — Appendix Figure Source Data [file 44321_2025_362_MOESM12_ESM.zip › S2-facs-dot-plots/No.5/DAY3-W3.jpg]

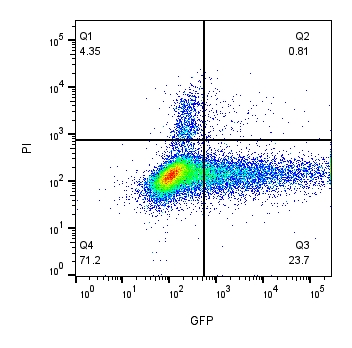

Supplement: Supplementary file 12 — Appendix Figure Source Data [file 44321_2025_362_MOESM12_ESM.zip › S2-facs-dot-plots/No.2/day3-r6c.jpg]

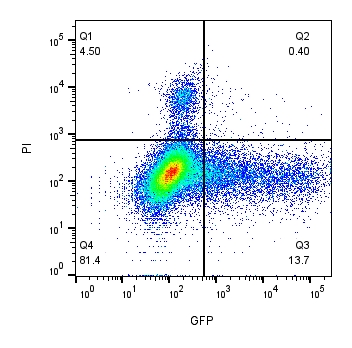

Supplement: Supplementary file 12 — Appendix Figure Source Data [file 44321_2025_362_MOESM12_ESM.zip › S2-facs-dot-plots/No.2/day1-wt+r6c.jpg]

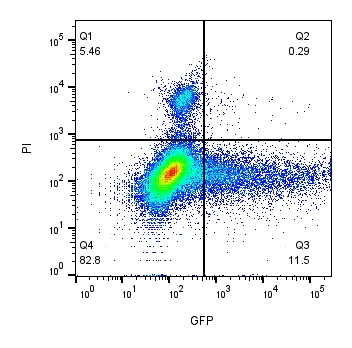

Supplement: Supplementary file 12 — Appendix Figure Source Data [file 44321_2025_362_MOESM12_ESM.zip › S2-facs-dot-plots/No.2/day1-r6c.jpg]

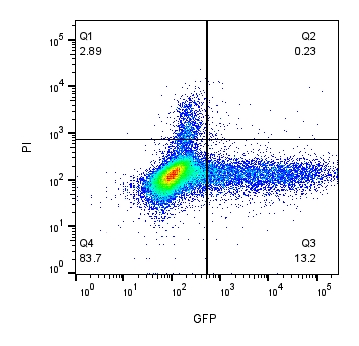

Supplement: Supplementary file 12 — Appendix Figure Source Data [file 44321_2025_362_MOESM12_ESM.zip › S2-facs-dot-plots/No.2/day3-empty.jpg]

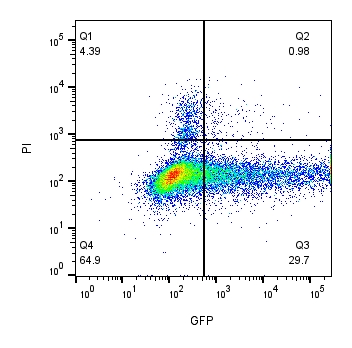

Supplement: Supplementary file 12 — Appendix Figure Source Data [file 44321_2025_362_MOESM12_ESM.zip › S2-facs-dot-plots/No.2/day3-wt+r6c.jpg]

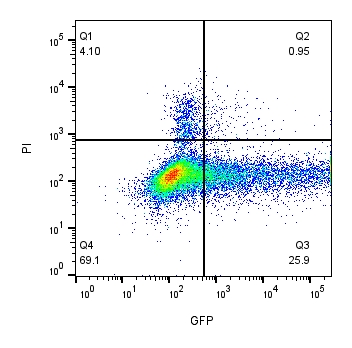

Supplement: Supplementary file 12 — Appendix Figure Source Data [file 44321_2025_362_MOESM12_ESM.zip › S2-facs-dot-plots/No.2/day3-wt.jpg]

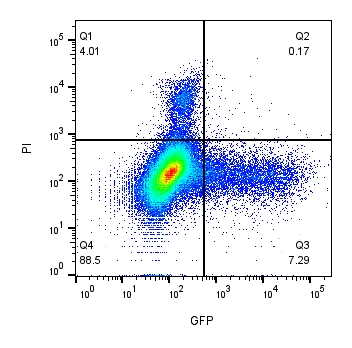

Supplement: Supplementary file 12 — Appendix Figure Source Data [file 44321_2025_362_MOESM12_ESM.zip › S2-facs-dot-plots/No.2/day1-empty.jpg]

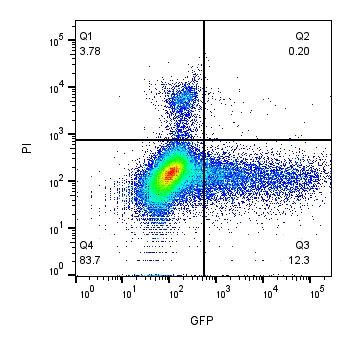

Supplement: Supplementary file 12 — Appendix Figure Source Data [file 44321_2025_362_MOESM12_ESM.zip › S2-facs-dot-plots/No.2/day1-wt.jpg]
