# Supplementary material for: A new form of diabetes caused by INS mutations defined by zygosity, stem cell and population data
Source: EMBO Mol Med. 2026 Jan 3;18(2):620–45. doi: 10.1038/s44321-025-00362-9 (PMC12905373; doi:10.1038/s44321-025-00362-9)
Supplement: Supplementary file 13 — Expanded View Figures [file 44321_2025_362_MOESM13_ESM.pdf]

Expanded View Figures

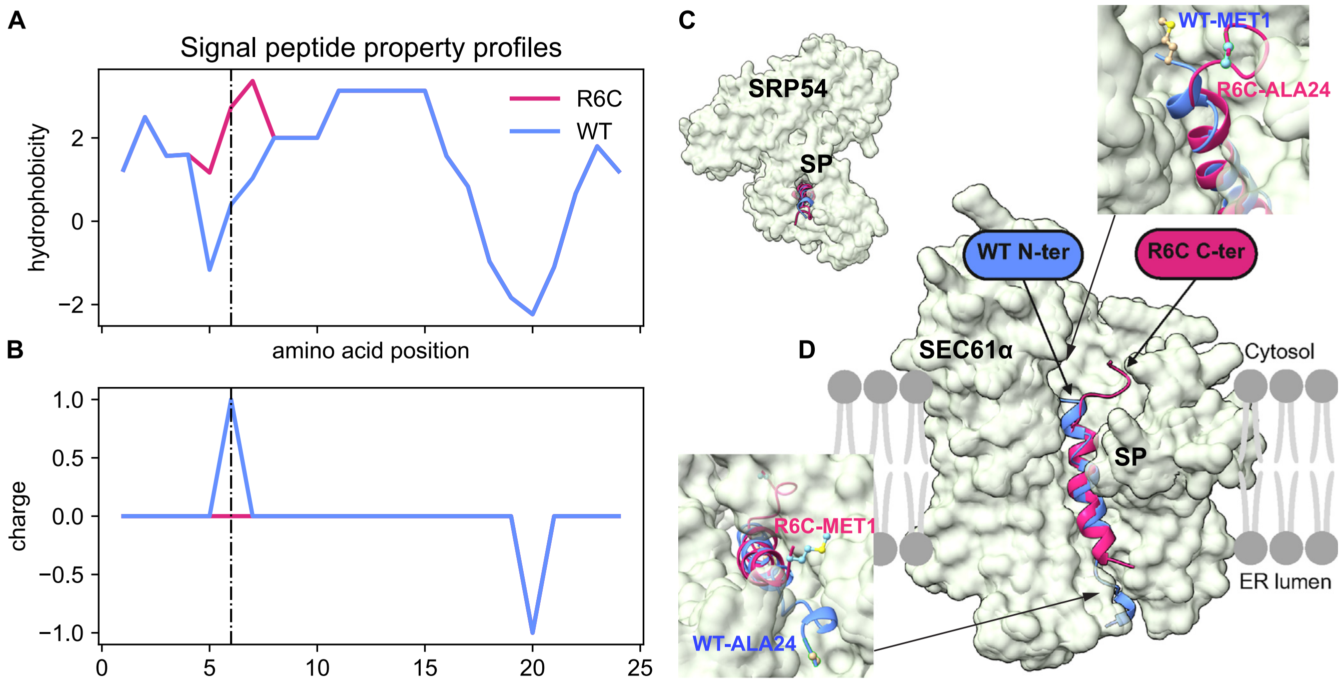

Figure EV1. Comparison of wild-type and mutant signal peptide properties and interactions with SRP54 and SEC61 complex.

Plotting and modeling used the first 24 amino acids of wildtype (WT, blue) or R6C (pink) preproinsulin. Hydrophobicity (**A**) and charge (**B**) profiles of the signal peptide (SP) for WT and R6C sequences. The dashed line indicates the position of the R6C substitution. (**C**) Structural model of aligned WT and R6C signal peptide bound to the SRP54 subunit, showing preserved orientation and overall binding. (**D**) Structural model of aligned WT and R6C signal peptide inserted into the SEC61α translocon, following an opposite insertion path into the ER membrane. Popped out window shows zoomed-in structure of cytosolic and lumen side of the ER, highlighting the first (1) and last (24) amino acids of either WT or R6C SP. Arabic number indicates the number of amino acids within the SP WT or R6C; MET methionine, ALA alanine. Scoring of both model details are listed in Appendix Table S4. Source data are available online for this figure.

 © The Author(s)

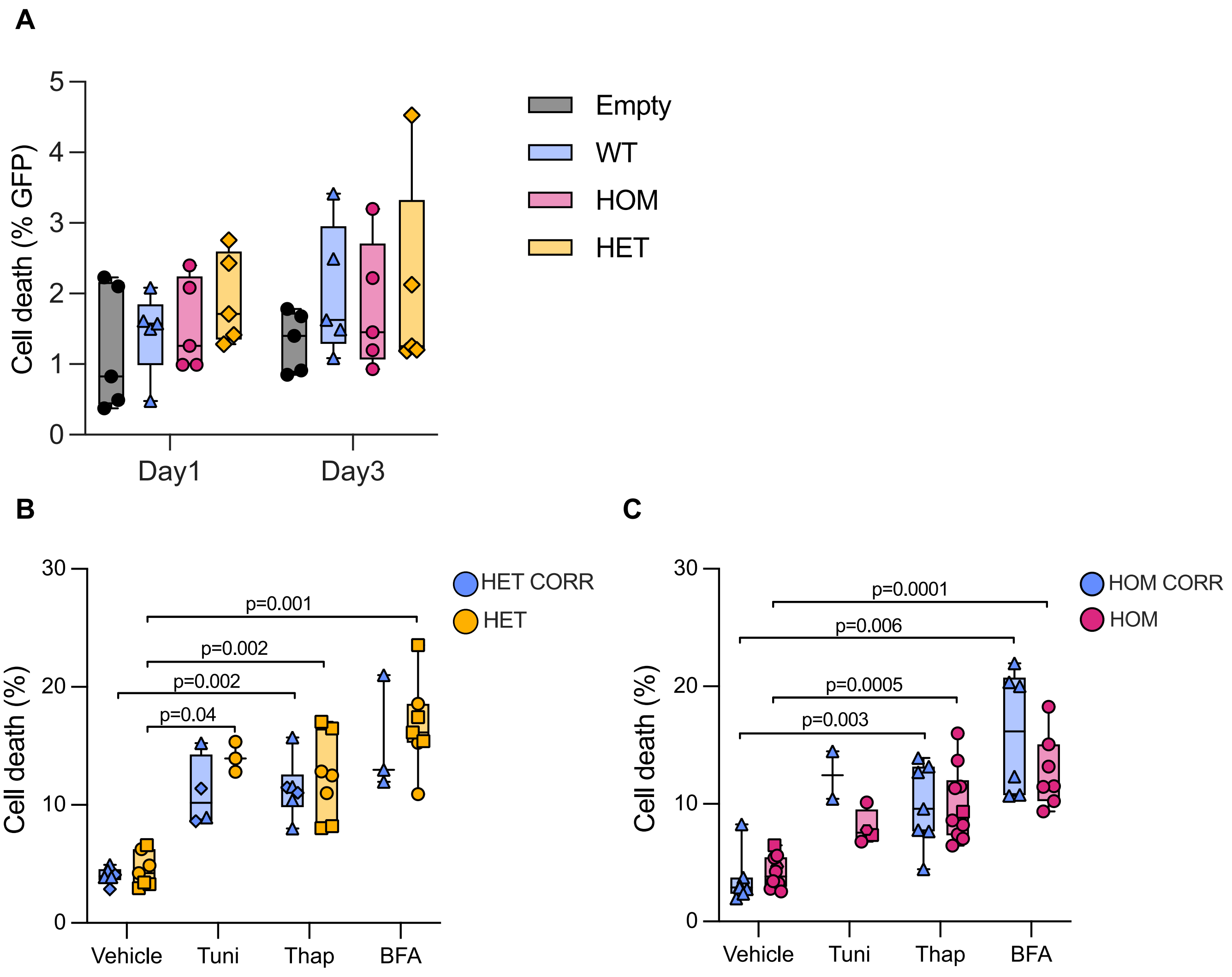

Figure EV2. ***INS* R6C does not induce β-cell death.**

(**A**) EndoC-βH1 INS-knockout cells were transfected with plasmids expressing wild-type (blue, WT) insulin, 100% R6C insulin (pink, R6C), 50% R6C + 50% wild-type insulin (yellow, HET), or GFP only (black, Empty) for 1 or 3 days. Cells were stained with propidium iodide (PI) and sorted for GFP expression and PI staining. Quantification of β cell death (%) as measured by percentage of double positivity for GFP and PI in total GFP positivity, $n = 5$. (**B**) Heterozygous R6C (HET, yellow) and isogenic corrected (HET CORR, blue) iPSCs and (**C**) homozygous (HOM, pink) and isogenic corrected (HOM CORR, blue) stage 7 iPSC-islets were exposed to synthetic ER stressors (Brefeldin A: BFA, 0.025 µg/mL, 24 h, thapsigargin: Thap, 1 µM, 48 h, or tunicamycin: Tuni, 5 µg/mL, 48 h) and cell death was assessed (%). Sample sizes (*n*) for each condition (HET CORR vs. HET; HOM CORR vs. HOM) were: Vehicle (DMSO), 6 vs. 7 and 7 vs. 10; BFA, 3 vs. 7 and 6 vs. 7; Thap, 6 vs. 7 and 7 vs. 10; Tuni, 4 vs. 3 and 2 vs. 4. Mixed-effects analysis with Tukey correction for multiple comparisons. In box plots, the median of independent experiments is shown by a horizontal line; 25th and 75th percentiles are at the bottom and top of the boxes; whiskers represent the minimum and maximum values. Source data are available online for this figure.

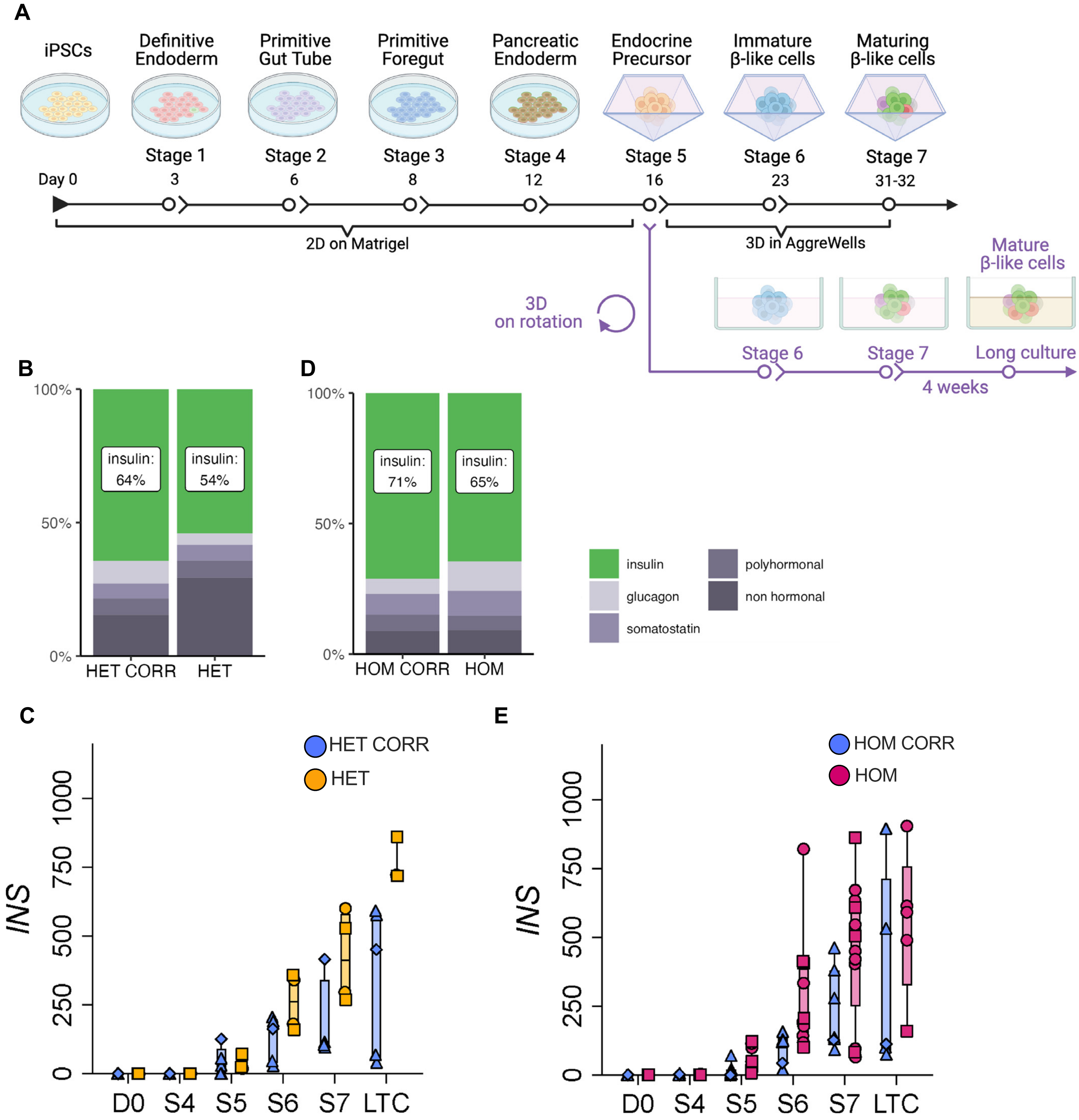

Figure EV3. R6C and corrected iPSCs differentiate into β cells.

(**A**) Scheme illustrating the β cell differentiation protocol. (**B**) Heterozygous and corrected iPSC-islet cell composition as stained for insulin, glucagon, and somatostatin. HET CORR $n = 6$, HET $n = 7$. (**C**) *INS* mRNA expression in heterozygous R6C and corrected iPSC lines at iPSC stage (D0) and along differentiation stages to long-term culture (LTC). Data were normalized to the geometric mean of reference genes β-Actin and VAPA. HET CORR $n = 5$, HET $n = 4$. (**D**) Homozygous and corrected iPSC-islet cell composition as stained for insulin, glucagon, and somatostatin. HOM CORR $n = 6$, HOM $n = 14$. (**E**) *INS* mRNA expression in homozygous R6C and corrected iPSC lines at D0 and along differentiation stages to LTC. Data were normalized to the geometric mean of reference genes β-Actin and VAPA. HOM CORR $n = 8$, HOM $n = 15$. In box plots, the median of independent experiments is shown by a horizontal line; 25th and 75th percentiles are at the bottom and top of the boxes; whiskers represent the minimum and maximum values. Source data are available online for this figure.

 © The Author(s)
